# Analysis of the brain palmitoyl-proteome using both acyl-biotin exchange and acyl-resin-assisted capture methods

**DOI:** 10.1038/s41598-017-03562-7

**Published:** 2017-06-12

**Authors:** Matthew J. Edmonds, Bethany Geary, Mary K. Doherty, Alan Morgan

**Affiliations:** 1 Department of Cellular and Molecular Physiology, Institute of Translational Medicine, University of Liverpool, Crown St., Liverpool L69 3BX UK; 2 Division of Health Research, University of the Highlands and Islands, Centre for Health Science, Old Perth Road, Inverness, IV2 3JH UK

## Abstract

Palmitoylation is a reversible post-translational protein modification in which palmitic acid is added to cysteine residues, allowing association with different cellular membranes and subdomains. Recently, techniques have been developed to identify palmitoylation on a proteome-wide scale in order to reveal the full cellular complement of palmitoylated proteins. However, in the studies reported to date, there is considerable variation between the sets of identified palmitoyl-proteins and so there remains some uncertainty over what constitutes the definitive complement of palmitoylated proteins even in well-studied tissues such as brain. To address this issue, we used both acyl-biotin exchange and acyl-resin-assisted capture approaches using rat brain as a common protein source. The palmitoyl-proteins identified from each method by mass spectrometry were then compared with each other and previously published studies. There was generally good agreement between the two methods, although many identifications were unique to one method, indicating that at least some of the variability in published palmitoyl proteomes is due to methodological differences. By combining our new data with previous publications using mammalian cells/tissues, we propose a high confidence set of *bona fide* palmitoylated proteins in brain and provide a resource to help researchers prioritise candidate palmitoyl-proteins for investigation.

## Introduction

Palmitoylation, specifically *S*-palmitoylation, describes the post-translational modification of proteins with the 16-carbon fatty acid palmitate. Unlike other lipid modifications of proteins, palmitoylation is reversible, and the enzymes involved were discovered relatively recently^1–4^. The formation of its thioester link to cysteine residues is catalysed by palmitoyl acyl-transferases (PATs), also known as DHHCs after their conserved active site aspartic acid-histidine-histidine-cysteine motif, and the cleavage of palmitate is catalysed by de-palmitoylating enzymes^1, 5, 6^. Palmitoylation occurs on diverse substrates, both soluble and transmembrane, in a variety of cell types and tissues. As such, it is important in the regulation of many cellular processes, including Ras signalling and synaptic plasticity, and altered palmitoylation is associated with various human diseases, notably neurological disorders^7–9^.

A variety of different methods have been developed for studying protein palmitoylation. The classical technique of labelling with [3H]-palmitate, although powerful, has a number of drawbacks. For example, autoradiograph exposure times of weeks or months are not uncommon and purification of the individual protein of interest is required^10^. Problems may arise from metabolic conversion of radiolabelled palmitate, for example by β-oxidation or further elongation of the fatty acid. In addition, the efficiency of labelling is dependent on the penetration of labelled palmitate into the cells, its ratio to unlabelled palmitate and the rate of palmitoylation turnover on the individual protein. Finally, the lack of sensitivity of [3H]-palmitate labelling means that proteins expressed at a low level, such as receptors or ion channels, may not be detected^11^.

Clearly, [3H]-palmitate labelling is unsuitable for unbiased identification of palmitoylation on a proteome-wide scale. Three techniques have emerged more recently for purifying all palmitoylated proteins from extracts such as cell lysates or tissue homogenates. The first is acyl-biotin exchange (ABE), which was originally developed in response to the lack of sensitivity in existing methods and their restriction to living cells^11, 12^. The principle of ABE derives from the labile nature of the thioester bond between palmitate and the modified cysteine residue. This can be easily cleaved using neutral hydroxylamine (HA)^13^, revealing a free sulfhydryl group on the cysteine residue which can be labelled with a variety of constructs^11^. The selectivity of this method for palmitoylation depends on prior blocking of any existing sulfhydryl groups on unmodified cysteine residues, which can be performed using the thioreactive compounds N-ethylmaleimide (NEM) or methyl methanethiosulphonate (MMTS). The negative control is identically treated except for the use of Tris.HCl in place of HA. Palmitoylation can then be inferred by the enrichment of a protein in the HA-treated sample relative to the Tris control.

The applicability of ABE to lysates to allow characterisation of *in vivo* palmitoylation was first demonstrated in *Saccharomyces cerevisiae* to characterise its palmitoylated membrane proteins^12^. This group took the ABE method developed by Drisdel and Green – labelling using a biotin construct and purifying all labelled proteins on streptavidin agarose – and extended it by using multi-dimensional protein identification technology (MudPIT) mass spectrometry to identify the proteins. The power of this method was demonstrated by the identification of 12 of the 15 previously known palmitoyl-proteins along with 35 new candidates. Such an analysis would not have been possible using radiolabelling approaches. A similar study soon followed using mammalian tissue, specifically rat cultured embryonic neurons and brain-derived synaptosomes^14^. In this case 68 known palmitoyl-proteins were identified along with 113 previously unknown high confidence candidates. ABE has since been applied to several mammalian cell types, including endothelial cells^15^, platelets^16^, macrophages^17^, and B lymphocytes^18^ (Supplementary Table 1). The palmitoyl-proteins present in lipid rafts have been characterised in human prostate cancer cells by ABE^19^, and the method has recently been used for the first characterisation of palmitoylation in *Arabidopsis thaliana* ^20^.

Acyl-resin-assisted capture (acyl-RAC) is an alternative technique which has been described recently^21^ and was derived from a modification of the biotin switch assay used to study *S*-nitrosylation^22^. It works in a similar way to ABE but shortens the protocol by pulling down the HA-treated proteins directly using a thioreactive sepharose. This has the advantage of reducing the number of steps and reactions, which may enable palmitoyl-proteins to be more efficiently purified and therefore increase the sensitivity over ABE. The proof-of-principle study was performed using bovine brain membrane proteins, showing detection of the palmitoyl-proteins Gαz and growth-associated protein of 43 kDa (GAP43; neuromodulin) by immunoblotting but not synaptophysin, which is known not to be palmitoylated^21^. The method was extended by examining which cysteine residues were palmitoylated in several proteins. Experimental and control HEK293 cells were labelled with iTRAQ reporter tags followed by tryptic digestion for mass spectrometry whilst the proteins were still bound to the resin. Both previously known and novel palmitoylation sites were discovered using this approach.

The third approach is a metabolic labelling method using palmitate analogues such as 17-ODYA/alk-16 and therefore represents a palmitate-centric, rather than a cysteine-centric approach. The proteins labelled with these reagents are reacted with azide reporters using copper(I) catalyzed azide alkyne cycloaddition (click) chemistry^23^, allowing overall visualisation by fluorescence in polyacrylamide gels and purification for mass spectrometric analysis. This method is particularly powerful for investigating palmitoylation dynamics, but as labelling of living cells is required it is not suitable for studying palmitoyl proteomics on animal-derived tissues such as brain.

Although a number of proteome-wide studies of palmitoylation have been performed over recent years, there is a surprisingly large amount of variation between the sets of palmitoyl proteins identified in these studies. Some proteins, such as cysteine string protein (CSP; encoded by the *DNAJC5* gene), are reported in multiple studies; whereas many others are identified in only a single publication (Supplementary Figure 1, Supplementary File 1). It is unclear if this observed variability is mainly due to methodological differences or to the use of different cell/tissue sources. Furthermore, there are no published data evaluating different palmitoyl-proteomic methods in head-to-head tests. To address these issues, we have optimised both ABE and acyl-RAC approaches using rat brain homogenate as a common protein source. Brain was chosen because aberrant palmitoylation has been associated with various neurological and psychiatric disorders, therefore a more definitive assessment of which brain proteins are palmitoylated may be of medical relevance. The palmitoyl-proteins identified from each method by mass spectrometry were then compared with each other and previously published studies. Palmitoyl-proteins were identified at a similar level to previous studies. There was a good level of agreement between the two methods, although many identifications were unique to one method, suggesting that at least some of the variability in published palmitoyl proteomes is due to methodological differences.

## Results

**Figure 1:**
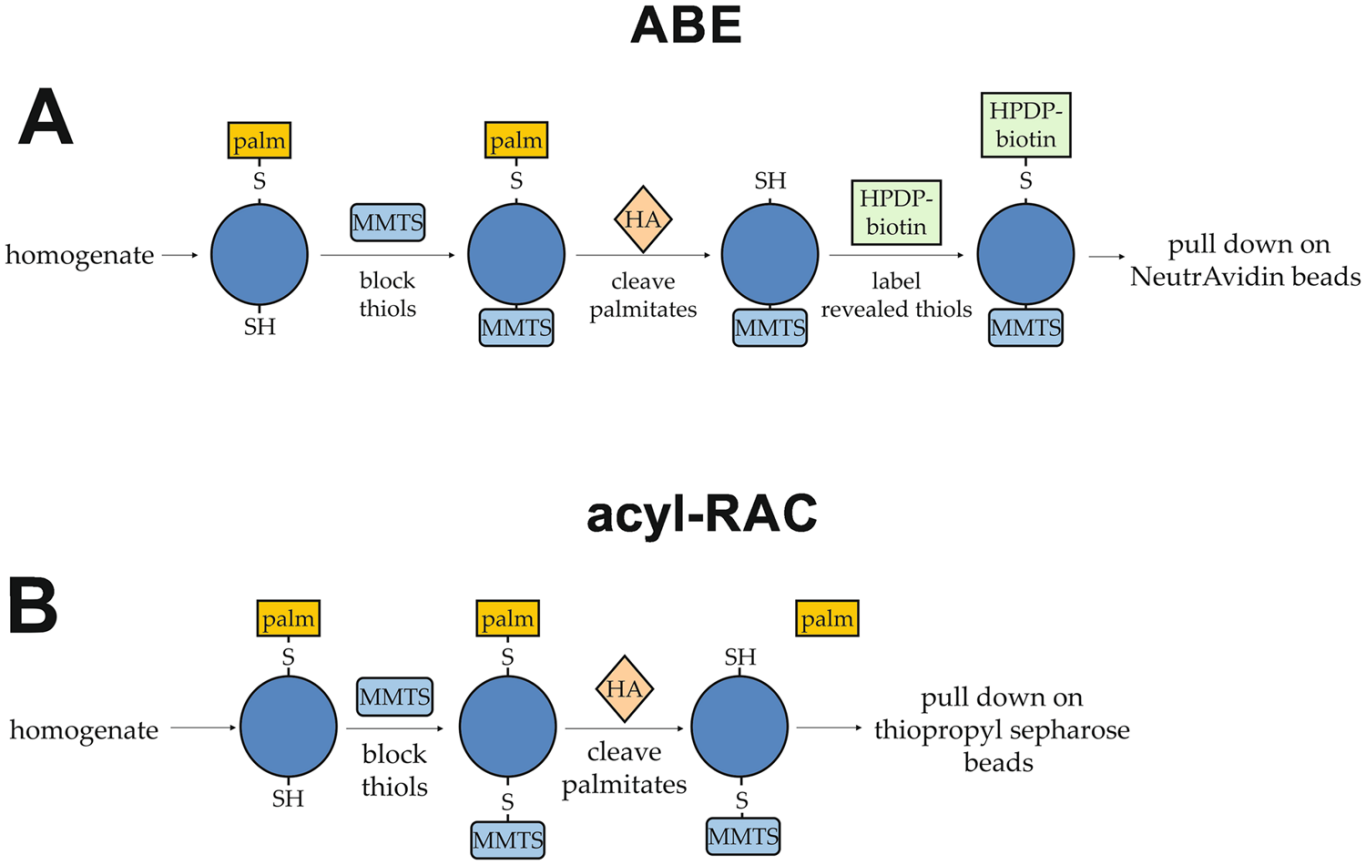
Principles of the acyl-biotin exchange and acyl-resin-assisted capture methods. The procedures for purification of palmitoyl-proteins from an extract using (**A**) acyl-biotin exchange and (**B**) acyl-resin-assisted capture are shown in cartoon form. Biotin-HPDP, N-[6-(biotinamido)hexyl]-3′-(2′-pyridyldithio)propionamide; HA, hydroxylamine; MMTS, methyl methanethiosulphonate; palm, palmitate.

There are several published palmitoyl-proteome analyses in various organisms and tissues^12, 14–21, 24–33^ (Supplementary Table 1). However, these data are primarily hidden in dense supplementary figures, making it difficult to compare them effectively. Recent research has curated identifications from mammalian studies with respect to gene ontology and disease association^34^, and the SwissPalm database collates all reported palmitoyl-proteins and palmitoylation sites in all species^35^. However, 61.7% (1123 of 1819) of palmitoyl-proteins identified at high confidence in these studies have not been replicated in others (Supplementary Figure 1). This may reflect the considerable variation in the methods used, both between ABE, acyl-RAC and click chemistry but also with the subtleties of the specific chemicals, extraction conditions and reaction times used. In addition, cell/tissue-specific protein expression can make direct comparisons between different model systems difficult. In order to investigate the dependence of the palmitoyl-proteins identified on the method used, and to better define the brain palmitoyl-proteome, we used the cysteine-centric methods ABE and acyl-RAC (Fig. 1) on brain tissue from adult female Sprague Dawley rats. We chose not to assess click chemistry methods due to the necessity of metabolic labelling in the living animals.

### Optimisation of ABE and acyl-RAC

The optimum reaction conditions for these techniques were first assessed. The ABE and acyl-RAC methods both use HA to cleave the labile thioester bond between cysteine residues and palmitate groups (Fig. 1). However, there have also been reports of disruption of this bond using alternative treatments, such as high pH and dithiothreitol (DTT)^36^. In light of this, free thiols on unmodified cysteine residues in proteins in rat brain homogenate were blocked using MMTS and then treated with HA, DTT or high pH. Samples were immunoblotted for cysteine string protein (CSP) because removal of its palmitate groups gives a clearly detectable shift in molecular weight from approximately 29 kDa to 22 kDa^37^. This shift was only seen with treatment with HA (Supplementary Figure 2A). A timecourse was employed to determine whether an overnight treatment with HA was necessary for complete depalmitoylation of a sample. Rat brain homogenate was treated with MMTS then incubated with HA for various lengths of time before removal of HA by methanol precipitation to terminate the reaction. Samples were again immunoblotted for CSP. A gradual downward shift in the molecular weight of CSP with increasing length of HA treatment was seen with complete depalmitoylation only occurring with an overnight incubation, confirming that this is required (Supplementary Figure 2B).

**Figure 2:**
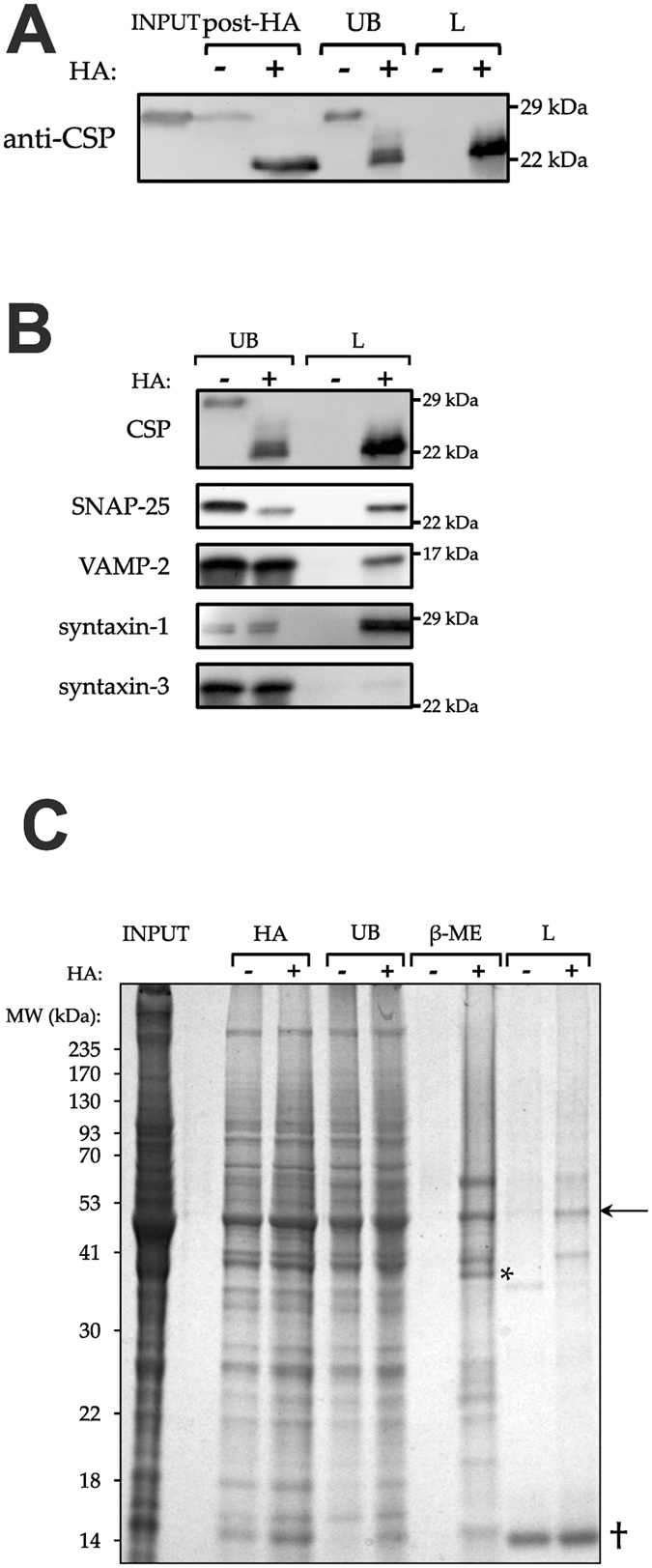
ABE selectively purifies palmitoylated proteins. (**A**) Samples from an ABE experiment were subjected to Western blotting and probed against CSP. Post-HA was taken after the HA treatment; unbound was the fraction that did not bind to NeutrAvidin-coated beads; the elution of proteins from the beads was performed with 4X Laemmli buffer. The mass shift from 29 kDa to 22 kDa shows depalmitoylation of CSP by HA, and that this is required for binding to the beads. (**B**) After ABE, unbound proteins and proteins eluted from the NeutrAvidin beads using Laemmli were subjected to Western blotting. Western blots were probed using antibodies against the proteins shown. Syntaxin-3 is a negative control containing no cysteine residues. (**C**) ABE was performed with a gentler 1% β-ME elution incorporated. SDS-PAGE gels were stained using Coomassie blue. Purification of palmitoyl-proteins is seen by an enrichment in the +HA samples compared with the -HA samples in the β-ME elutions. Examples are shown by symbols: an arrow shows bands enriched in the +HA samples with both β-ME and subsequent Laemmli elution; an asterisk shows bands enriched in the +HA samples only in the β-ME elution; a dagger shows non-specific bands which are present in the Laemmli elutions only. Cropped images are displayed here, but full-length gels and blots are shown in Supplementary Figure 6. β-ME, β-mercaptoethanol; CSP, cysteine string protein; L, Laemmli elution; SNAP-25, synaptosomal-associated protein of 25 kDa; UB, unbound; VAMP-2, vesicle-associated membrane protein 2.

Having established depalmitoylation conditions, a full scale ABE protocol was carried out on rat brain homogenate. Samples were taken after the HA treatment, from the solution after incubation with NeutrAvidin-conjugated beads (UB; unbound) and after eluting from the beads by boiling in 4X Laemmli solubilisation buffer (L) and were all immunoblotted for CSP (Fig. 2A). All the HA-treated samples showed the characteristic mass shift of depalmitoylated CSP. If a protein is palmitoylated, it should be present in the final eluate from the HA-treated sample but not the control sample. This was the case with CSP, which was specifically detected in the +HA eluate. There was still some CSP retained in the unbound +HA sample however, suggesting incomplete binding to the beads. As a result of this, the quantity of beads used in the final binding was doubled for subsequent experiments. The unbound and eluted samples were also probed for a panel of other proteins (Fig. 2B). Along with CSP, the known palmitoyl-proteins synaptosomal-associated protein of 25 kDa (SNAP-25)^38^ and vesicle associated membrane protein 2 (VAMP-2; synaptobrevin-2)^39^ were specifically found in the +HA elution. Syntaxin-1 was found to be palmitoylated in the first proteomic screen of mammalian material using ABE^14^ and was also detected here. Complexes of soluble NEM-sensitive fusion protein (NSF)-attachment protein (SNAP) receptor (SNARE) proteins have been shown to be resistant to denaturation by SDS even at temperatures up to 80 °C^40^. As SDS is used in ABE, we were concerned that detection of syntaxin-1 might be a false positive if it was retained in SNARE complexes with SNAP-25. Syntaxin-3 contains no cysteine residues and so cannot be palmitoylated. If SNARE complexes are pulled down then syntaxin-3 should also be detected in this way, as it too forms SDS-resistant complexes with SNAP-25 and VAMP. No signal was seen from syntaxin-3 in the eluates, confirming the specificity of the ABE procedure (Fig. 2B).

The eluates from this initial ABE experiment were analysed using mass spectrometry, and after curation of the results there were many protein identifications which seemed likely false positives, such as subunits of haemoglobin and actin at high abundance (data not shown). To try to combat this, a gentler elution condition was used. Instead of boiling in 4X Laemmli, the beads were incubated in a buffer containing 1% β-mercaptoethanol (β-ME) at 37 °C for an hour with occasional agitation. The beads were also boiled in 4X Laemmli after this elution to get a measure of what proportion of the genuine palmitoyl-proteins was being eluted by β-ME. To visualise this, samples were subjected to SDS-PAGE and the gel stained with Coomassie blue (Fig. 2C). At the post-HA treatment and unbound stages, there were roughly equal amounts of protein in both the experimental and control samples. The β-ME elution showed a clear enrichment of proteins in the HA-treated lane over the control lane (Fig. 2C, asterisk). Whilst enrichment can also be seen in the Laemmli eluates, it was to a much lesser degree and there are many non-specific bands which appear strongly in both HA and control treatments (Fig. 2C, dagger).

**Figure 3:**
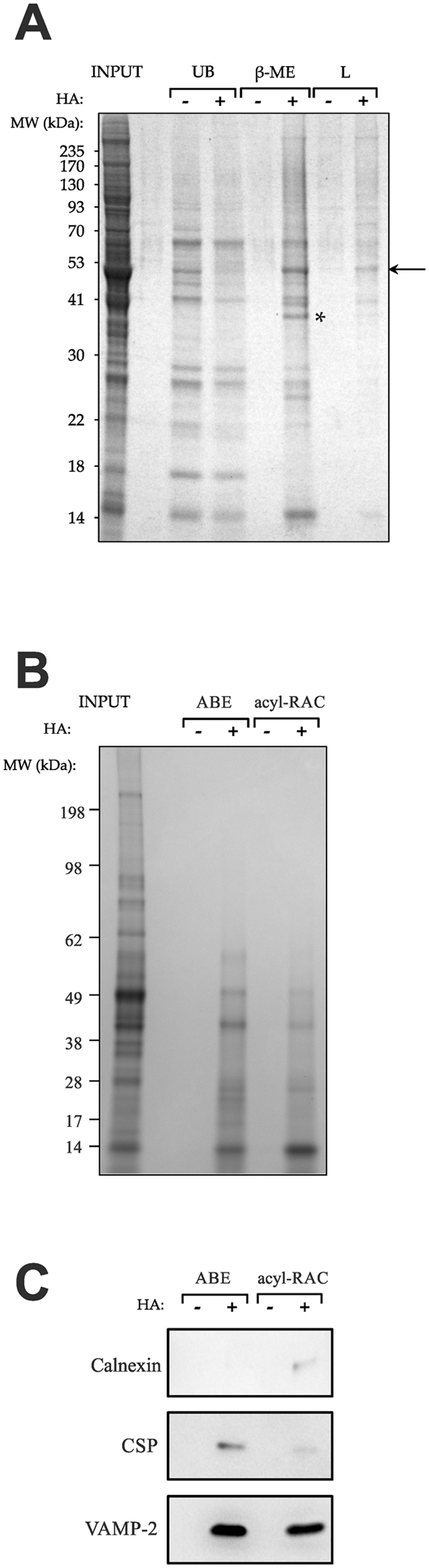
Comparison of the ABE and acyl-RAC methods. (**A**) Acyl-RAC was performed and SDS-PAGE gels stained with Coomassie blue. Specifically purified palmitoyl-proteins can be seen: an arrow shows bands enriched in the +HA samples with both β-ME and subsequent Laemmli elution; an asterisk shows bands enriched in the +HA samples only in the β-ME elution. (**B**,**C**) Samples from ABE and acyl-RAC experiments were run in parallel on SDS-PAGE and stained with Coomassie blue (**B**) or western blotted (**C**) using antibodies against calnexin, CSP and VAMP-2. Cropped images are displayed here, but full-length gels and blots are shown in Supplementary Figure 6. β-ME, β-mercaptoethanol; CSP, cysteine string protein; VAMP-2, vesicle-associated membrane protein 2.

The alternative method of acyl-RAC was also used to assess the palmitoyl-proteome of rat brain material. Our initial attempt using rat brain homogenate and the published control treatment of 2 M NaCl^21^ was unsuccessful. However, changing the control treatment to 2 M Tris.HCl, as in ABE, solved this problem. Stained gels of the acyl-RAC experiment show specific bands in the HA-treated β-ME elution (Fig. 3A; asterisk), as in ABE above. There is no post-HA sample in the acyl-RAC protocol because the HA treatment and binding to the resin occur in the same step (Fig. 1). Thus, similar reaction conditions for each technique were determined (Supplementary Figures 3,4) and Coomassie blue staining of β-ME elutions from ABE and acyl-RAC protocols run side-by-side on SDS-PAGE produced apparently similar profiles of major eluted proteins (Fig. 3B).

### Mass spectrometry analysis of eluates

Eluates from three independent ABE and acyl-RAC experiments on rat brain homogenate were subjected to mass spectrometry. The data from all three biological replicates were analysed together and identifications made using MaxQuant with a false discovery rate (FDR) of 1%. This yielded protein identifications for both the experimental (HA-treated) and control (Tris.HCl-treated) conditions for each method from which identifications from only one unique peptide were removed (Supplementary File 2). Proteins that appeared in both the control and experimental list for the same technique were removed from the HA-treated list to give a stringent list of palmitoyl-proteins that were identified using each method.

Analysis of ABE yielded 241 identifications (Supplementary Table 2) and acyl-RAC yielded 144 identifications (Supplementary Table 3). These numbers are a similar level to those from previous studies (Supplementary Table 1). 61 proteins were found in common between the ABE and acyl-RAC analyses, which can be considered the highest confidence protein identifications (Table 1). This corresponds to 25% (61 of 241) of the ABE identifications and 42% (61 of 144) of the acyl-RAC identifications. It was notable that most proteins identified using one method were not found using the other, including a number of well-known palmitoyl-proteins. To investigate if this was due to inherent substrate selectivity differences in the ABE/acyl-RAC methods or to false negatives introduced as a result of our stringent mass spectrometry cut-offs, we performed western blots on several differentially identified proteins (Fig. 3C). Calnexin was detected by immunoblotting in the acyl-RAC HA eluate but not the ABE HA eluate (Fig. 3C), thus mirroring its detection by mass spectrometry using acyl-RAC, but not ABE. Conversely, CSP, which was identified by ABE, but not by acyl-RAC, was much more enriched in the ABE HA eluate compared to the acyl-RAC HA eluate, consistent with the mass spectrometry results. In contrast, VAMP-2/synaptobrevin 2 was readily detected in both ABE and acyl-RAC HA eluates by immunoblotting (Fig. 3C), whereas it was only positively identified by mass spectrometry using ABE due to it being detected in the control Tris eluate using acyl-RAC. This may suggest that the ABE method is more specific but that acyl-RAC is more sensitive; a notion supported by the observation that roughly similar total numbers of peptides were detected in both HA experiments (acyl-RAC HA – 2621; ABE HA – 1968), but considerably more peptides were identified in the control Tris elutions for acyl-Rac (2170) compared to ABE (300) (Supplementary File 3). Thus, although mass spectrometry is a powerful method for identification of the palmitoyl proteome, failure to detect a protein should not be taken to mean that it is definitively not palmitoylated, unless multiple methods are employed.

**Table 1:** Protein identifications from both ABE and acyl-RAC using rat brain homogenate.

*Gene*	Protein	Previously identified?
*A1bg*	α-1B-glycoprotein	
*Acp1*	low molecular weight phosphotyrosine protein phosphatase, isoform 1	
*Ak2*	adenylate kinase 2, mitochondrial isoform 1	✓
*Akr1b1*	aldose reductase	
*Apoa1bp*	NAD(P)H-hydrate epimerase	✓
*Aspa*	aspartocyalase	
*Atp1b2*	β2 subunit of sodium/potassium-ATPase	✓
*Atp5o*	ATP synthase subunit O, mitochondrial	✓
*Atp6v1g2*	ATPase, H + transporting, lysosomal, V1 subunit G2	
*Cadps*	calcium-dependent secretion activator 1	✓
*Celf2*	CUG triplet repeat RNA-binding protein 2 (CUGBP2)	
*Ckmt1*	creatine kinase U-type, mitochondrial	✓
*Ddah1*	N(G), N(G)-dimethylarginine dimethylaminohydrolase 1	
*Eef1b2*	eukaryotic translation elongation factor 1 β2	
*Erp29*	endoplasmic reticulum protein ERp29	
*Fam49b*	family with sequence similarity 49, member B (also called 0910001A06Rik)	✓
*Fetub*	fetuin-B	
*Gap43*	neuromodulin	✓
*Glod4*	glyoxolase domain-containing protein 4	
*Gnao1*	Gαo G protein subunit	✓
*Hdhd2*	haloacid dehalogenase-like hydrolase domain-containing 2	
*Hspa9*	heat shock 70 kDa protein 9	✓
*Itpa*	inosine triphosphatase	
*Map1lc3a*	microtubule-associated proteins 1 A/1B light chain 3 A	
*Mog*	myelin-oligodendrocyte glycoprotein	✓
*Nap1l4*	nucleosome assembly protein 1-like 4	
*Napg*	N-ethylmaleimide-sensitive factor (NSF) attachment protein γ	
*Ndrg4*	brain development-related molecule 1	
*Nme2*	nucleoside diphosphate kinase B	✓
*Omg*	oligodendrocyte myelin glycoprotein	
*Pacs1*	phosphofurin acidic cluster sorting protein 1	
*Pacs2*	phosphofurin acidic cluster sorting protein 2	
*Pacsin1*	protein kinase C and casein kinase substrate in neurons 1	
*Pdia3*	protein disulphide-isomerase A3	✓
*Pdia6*	protein disulphide-isomerase A6	✓
*Pgls*	6-phosphogluconolactonase	
*Pitpna*	phosphatidylinositol transfer protein α	
*Pnpo*	pyridoxine-5-phosphate oxidase	
*Ppa1*	pyrophosphatase 1	✓
*Ranbp1*	RAN binding protein 1	✓
*Rap1gds1*	RAP1, GTP-GDP dissociation stimulator 1	
*Rbm28*	RNA binding motif protein 28	
*Rpl12*	60 S ribosomal protein L12	✓
*S100a16*	protein S100-A16	
*Sdhb*	succinate dehydrogenase [ubiquinone] iron-sulfur subunit, mitochondrial	✓
*Serpina1*	α1-antiproteinase	
*Serpina3k*	serine protease inhibitor A3K	
*Sfrs3*	splicing factor, arginine/serine-rich 3	
*Snap91*	clathrin coat assembly protein AP180 long isoform	✓
*Snx12*	sorting nexin 12	
*St13*	Hsc70-interacting protein	
*Stub1*	STIP1 homology and U-box containing protein 1	
*Tagln2*	transgelin-2	✓
*Tpt1*	translationally-controlled tumour protein	✓
*Trappc3*	trafficking protein particle complex 3 (Bet3)	✓
*Txn1*	thioredoxin	
*Txndc17*	thioredoxin-like 5	
*Ube2f*	NEDD8-conjugating enzyme E2 F	
*Ube2m*	ubiquitin-conjugating enzyme E2 M	✓
*Vapa*	vesicle-associated membrane protein-associated protein A	
*Vapb*	vesicle-associated membrane protein-associated protein B	

Proteins identified in both ABE and acyl-RAC are listed in alphabetical order of gene name. Proteins also identified in previous proteomic studies are indicated.

All high confidence protein identifications from previous screens using mammalian material were collated and compared with our mass spectrometry identifications (Supplementary File 1). Identifications using each method in this study show a higher proportion of previously identified palmitoyl-proteins than in all combined studies (Supplementary Figure 5A–C). We found 8.5% (165 of 1819) of previously identified palmitoyl-proteins in our dataset, and these accounted for 52% (125 of 241) of our identifications using ABE and 37% (53 of 144) with acyl-RAC (Supplementary Figure 5D). This relatively low overlap reflects what has been seen with other studies (Supplementary Figures 1 and 5) and underlines the fact that individual identifications from a single study cannot be taken at face value to mean a protein is (or is not) palmitoylated. Nevertheless, it is possible that our optimised ABE/acylRAC protocols also contribute to the new identifications reported here. In making an assessment of confidence in a palmitoyl-protein, the number of studies it is identified in should be taken into account. This can be easily found in Supplementary File 1. Our analysis indicates that only 38 out of 1988 proteins (1.9%) are identified in half or more of mammalian studies (Supplementary Figure 4C, Table 2). Many of these, including Ras proteins^41^, G protein subunits^42^ and the protein most frequently identified, calnexin^43^, have been independently confirmed as palmitoyl-proteins. These 38 should therefore be considered the highest confidence palmitoyl-proteins and could be prioritised for further investigation. In addition, cell-type-specific protein expression (Supplementary Table 1) should be taken into account. Indeed, many of the longest established palmitoyl-proteins, such as SNAP-25^38^ and VAMP-2^39^ are restricted to neuronal and regulated secretory cells and therefore only ubiquitously expressed homologues such as SNAP-23 and VAMP-3 are identifiable in the majority of palmitoyl-proteomic studies performed to date.

**Table 2:** Palmitoyl-proteins identified in at least half of mammalian proteomic studies.

Gene name	Protein name	No. of studies identified in
*Canx*	calnexin	15
*Gnai2*	Gαi2 G protein subunit	14
*Gnai3*	Gαi3 G protein subunit	13
*Gnaq*	Gαq G protein subunit	13
*Mtdh*	lysine-rich Ceacam1 co-isolated (Lyric)	13
*Nras*	N-Ras	13
*Snap23*	synaptosomal-associated protein of 23 kDa	13
*Flot2*	flotillin 2	12
*Gna13*	Gα13 G protein subunit	12
*Gnas*	Gαs G protein subunit short	12
*Hras*	H-Ras	12
*Pi4k2a*	phosphatidylinositol 4-kinase type IIα	12
*Rap2b*	Ras-related protein Rap-2b	12
*Scamp3*	secretory carrier membrane protein 3	12
*Flot1*	flotillin 1	11
*Gna11*	Gα11 G protein subunit	11
*Rap2c*	Ras-related protein Rap-2c	11
*Rras*	R-Ras	11
*Scamp1*	secretory carrier membrane protein 1	11
*Slc1a5*	neutral amino acid transporter B	11
*Stom*	stomatin; erythrocyte band 7 integral membrane protein	11
*Tfrc*	transferrin receptor	11
*Trappc3*	trafficking protein particle complex 3 (Bet3)	11
*Ckap4*	cytoskeleton-associated protein 4	10
*Fam49b*	family with sequence similarity 49, member B (also called 0910001A06Rik)	10
*Lamtor1*	late endosomal/lysosomal adaptor, MAPK and MTOR activator 1 (also called 2400001E08Rik, C11orf59)	10
*Plscr3*	phospholipid scramblase 3	10
*Rap2a*	Ras-related protein Rap-2a	10
*Stx6*	syntaxin 6	10
*Cd44*	CD44 antigen	9
*Dnajc5*	cysteine string protein (CSP)	9
*Ergic3*	endoplasmic reticulum-Golgi intermediate compartment protein 3	9
*Fyn*	Fyn non-receptor tyrosine kinase	9
*Lnpep*	leucyl-cysteinyl aminopeptidase isoform 1	9
*Scamp2*	secretory carrier membrane protein 2	9
*Scarb2*	scavanger receptor class B member 2; CD36 antigen-like 2; lysosomal integral membrane protein II (LIMP-II)	9
*Stx12*	syntaxin 12	9
*Vamp3*	vesicle-associated membrane protein 3; synaptobrevin 3	9

The 38 proteins listed here have been identified with high confidence in at least 9 out of 18 mammalian palmitoyl-proteome analyses (Supplementary File 1).

## Discussion

Palmitoylation is emerging as an important post-translational modification of proteins and this is reflected in the gradual increase in the sensitivity and scope of techniques available to detect it. Recently, proteomic techniques such as ABE and acyl-RAC have been developed to identify proteins that are robustly palmitoylated in tissues or whole organisms, as living cells are not required for labelling. However, these techniques have shown limited reproducibility, which may be down to differences in the palmitoyl-proteome of individual species or tissues or a more fundamental limitation of the techniques themselves. In order to better define the brain palmitoyl proteome and to investigate the contribution of the specific chemistry of ABE and acyl-RAC to the palmitoyl-proteins identified, we compared the outcome of both techniques on identical source tissue, rat brain.

ABE and acyl-RAC were initially optimised at the SDS-PAGE and Western blot level to eliminate as much variability between the protocols as possible. The eluates were analysed using mass spectrometry and were found to have a similar number of palmitoyl-proteins present to previous studies. Amongst the 61 identifications common to both methods (Table 1) were a number of well-known palmitoyl-proteins, including the G protein subunit Gα_o_^42^, GAP43/neuromodulin^44^ and trafficking protein particle complex 3 (TRAPPC3)/Bet3^45^. Notable also was thioredoxin, which is known to be palmitoylated^46^ but has not previously been identified in proteomic studies. The E2 enzymes for ubiquitin and neural precursor cell expressed, developmentally down-regulated 8 (NEDD8) are likely false positives due to their use of a cysteine thioester which would be detected by these assays^47, 48^. Of course these enzymes may indeed be palmitoylated, but assessment of this possibility would need careful investigation.

Whilst these proteomic identifications give a good guide as to potential palmitoyl-proteins, the results of individual studies of this scale should be interpreted with caution. In this study the majority of identifications from one method were not found using the other, including a number of *bona fide* palmitoyl-proteins. Amongst these were CSP^37^, syntaxin-1A and -1B^11, 14^, N-Ras^41^, Gα_q_^42^, acyl-protein thioesterase 2 (APT2)^49^ and cell division cycle 42 (Cdc42)^14^ that were identified by ABE, but not by acyl-RAC; and calnexin^43^ and APT1^49^ that were identified by acyl-RAC, but not by ABE.

What underlies the differential identifications we observed using these two methods, which are based on similar cysteine-centric chemistry ? At least part of the explanation is clearly due to inherent biochemical differences between the proteins that ABE and acyl-RAC enrich for, as shown here for calnexin and CSP (Fig. 3C). The reasons for this are not clear, but the string of 14 clustered cysteine residues within rat CSP would convert to a string of multivalent disulphide bonds that may make elution from the thiopropyl-Sepharose resin difficult. Consistent with this notion, SNAP-25, which has 4 clustered cysteine residues, was similarly detected by ABE but not by acyl-RAC, although in this case the protein was also detected in the Tris control elution. Indeed, some differential identifications are due to the stringent cut-off we applied, by eliminating protein identifications that were also detected in the Tris control. Data on all individual proteins can quickly be checked in Supplementary File 3, so that researchers can judge whether to pursue further study of their palmitoylation. Note, however, that adopting a previously published ratiometric approach for defining high-confidence palmitoyl proteins from brain^14^ (10-fold higher LFQ intensity of HA compared to Tris control) made little difference to the protein identifications for either ABE (279 proteins common to both filtering methods, with an additional 2 proteins unique to the ratiometric method) or acyl-RAC (241 proteins common to both filtering methods, with an additional 7 proteins unique to the ratiometric method) – see Supplementary File 4. We therefore conclude that the deliberately stringent and clear cut-off filter we applied does not have a major effect on protein identifications. Nevertheless, it is also important to bear in mind that the inherent variability of the multiple technical steps used in both the biochemical enrichment and mass spectrometry aspects of palmitoyl proteomics must inevitably account for some differential identifications.

Finally, the absence of a protein from such proteomic datasets does not mean it is definitively not palmitoylated, as in the example of thioredoxin noted above. Some well-known palmitoyl-proteins were not picked up by either method in our study, for example SNAP-23 and H-Ras. It is likely that this is mainly due to tissue-specific differences in expression, as SNAP-23 is expressed at low levels in brain compared to the very high expression level of the neuronal SNAP-25 isoform. However, it may also reflect a relative weakness of these methods to detect proteins undergoing rapid palmitate cycling, such as the Ras family members^50^, which may be better addressed using the metabolic labelling click-chemistry approach. Thus, this combination of the specificity of identifications to the method used, along with potential confounding factors from different species, strains and tissues, as well as inherent experimental variability, likely explains why the vast majority of identifications are only found in a single study.

## Conclusions

Despite the caveats discussed here, proteomics approaches do allow a large list of candidate palmitoyl-proteins to be produced, which is not possible using classical methods of studying palmitoylation. The variety of techniques used to gather these lists and the results from this study also suggests that the use of multiple techniques may give a better overall picture of the palmitoyl-proteome of a given tissue or organism. Many proteins are consistently detected, and indeed there are 38 proteins which have been detected in half or more of mammalian studies (Table 2, Supplementary Figure 5C,D). To help to address the variability between previous palmitoyl-proteome studies, we have added two new proteomic datasets and collated the mammalian identifications to provide an easily searchable and sortable list (Supplementary File 1). Searching this new resource in addition to the existing SwissPalm database^35^ will help researchers to assess the confidence of each putative palmitoyl-protein for their cell type of interest, and prioritise which candidates warrant further investigation.

## Methods

### Preparation of lysates

10 ml homogenisation buffer (HB; 0.32 M sucrose, 10 mM HEPES pH 7.4) containing one Complete Mini EDTA-free protease inhibitor (PI) tablet (Roche, Mannheim, Germany) was pre-chilled on ice. An adult female Sprague Dawley rat brain, snap-frozen (SeraLab, Barnet, UK), was thawed on ice in about 5 ml HB. The brain was cut into small pieces using dissection scissors on a glass plate then transferred to a specialised glass tube. The remaining HB was added and the tissue processed using an electric homogeniser until homogenous. The homogenate was spun at 3500 rcf at 4 °C for five minutes to remove debris. Sodium dodecyl sulphate (SDS) was added to the supernatant to 2% final concentration, rotated for 10–20 minutes at room temperature and spun at 18400 rcf at 4 °C for five minutes. The concentration of protein in a sample was determined using a Pierce® BCA Protein Assay Kit (Thermo Scientific, Rockford, IL, USA).

### Preliminary tests

To determine the best depalmitoylation agent, 100 μl 2 M methyl methanethiosulphonate (MMTS) stock (20 mM final concentration) was added to rat brain homogenate and made up to 10 ml with lysis buffer (LB; 150 mM NaCl, 50 mM Tris.HCl, 5 mM EDTA, pH 7.4) and a PI tablet was added. The mixture was incubated at room temperature for two hours. 500 μl samples were taken and treated with one of the following:

- 500 μl 2 M hydroxylamine (HA) pH 7.4 overnight at room temperature.
- 500 μl 40 mM dithiothreitol (DTT) for one hour at 37 °C.
- 500 μl 200 mM Tris pH 8.9 (high pH sample) for one hour at room temperature.

Samples were subjected to Western blotting against cysteine string protein (CSP); its depalmitoylation is indicated by a mass shift from approximately 29 to 22 kDa.

The time required for complete depalmitoylation of samples was also assessed. 100 μl rat brain homogenate was incubated with an equal volume of 2 M HA pH 7.4 or 2 M Tris.HCl pH 7.4 for 30 minutes up to overnight. Samples were subjected to three methanol precipitations (see ABE protocol, below) to remove HA. Western blotting was performed against CSP to determine the extent of depalmitoylation by mass shift.

### Acyl-biotin exchange

The protocol presented here represents the optimised method, based on protocols previously published in proteomic analyses^12, 14^. A schematic workflow of this method is shown in Supplementary Figure 2.

1. Rat brain homogenate was made up to 10 ml with LB and a PI tablet was added and a sample taken (“input”). 100 μl 2 M MMTS (20 mM final concentration) was added to the remainder and the sample incubated at room temperature for two hours.
2. The sample was split into three 15 ml Falcon tubes and a methanol precipitation performed three times to each as follows:
  - Three-times volume of −20 °C methanol was added and the tubes vortexed and spun at 3500 rcf, 4 °C for two minutes.
  - The supernatant was discarded and the pellet resuspended in 1 ml solubilisation buffer (SB; 4% SDS, 50 mM Tris.HCl, 5 mM EDTA, pH 7.4) and incubated at 37 °C, 220 rpm for 30 minutes.
  - The solution was made up to 4 ml total volume with LB +0.2% Triton X-100 (LB-T).
3. The combined volume was split into two 15 ml Falcons. 5 ml 2 M HA pH 7.4 was added to one tube and 5 ml 2 M Tris.HCl pH 7.4 to the other (1 M final concentration). 1.25 ml 4 mM EZ-link® N-[6-(biotinamido)hexyl]-3′-(2′-pyridyldithio)propionamide (biotin-HPDP) (Thermo Scientific, Rockford, IL, USA) stock was added to each tube (0.5 mM final concentration), both were made up to 10 ml with LB and a PI tablet was added. Tubes were incubated at room temperature on a rocker overnight.
4. The next day a sample was taken out (“post-HA”). The remainder of each treatment was divided into three tubes and three methanol precipitations performed. In the third methanol precipitation, 250 μl SB was used for solubilisation and made up to 10 ml with LB-T before incubating on a rocker at room temperature for 30 minutes.
5. Meanwhile, 600 μl NeutrAvidin® UltraLink® Resin (Thermo Scientific, Rockford, IL, USA) was put in a 15 ml Falcon for each sample. These were washed three times in 10 ml LB-T and spun at 3500 rcf, 4 °C for two minutes between washes.
6. The samples (from step 4) were spun at 3500 rcf, 4 °C for two minutes. Their supernatant was added to the washed beads and incubated at room temperature on a rocker for 90 minutes.
7. After spinning at 3500 rcf, 4 °C for two minutes a sample of the supernatant was taken (“unbound”). The pellet was washed four times with 10 ml LB-T +0.1% SDS, spinning at 3500 rcf, 4 °C for two minutes between washes.
8. Proteins were eluted in 3 ml LB-T +1% β-mercaptoethanol (β-ME) by incubating at 37 °C for one hour with occasional agitation. The samples were spun at 3500 rcf, 4 °C for two minutes. The supernatant was removed and treated as step 9 below. 1 ml 4X Laemmli buffer (8% SDS, 40% glycerol, 20% β-ME, 0.008% bromophenol blue, 0.25 M Tris.HCl, pH 6.8) was added to the bead pellet, which was resuspended and boiled – the supernatant of which was removed (“Laemmli elution”).
9. The supernatant from step 8 was split into 0.3 ml aliquots in 1.5 ml Eppendorfs. A three-times volume of −20 °C methanol was added, the tubes were vortexed and then spun at 10000 rpm, 4 °C for five minutes. The supernatant was discarded carefully. The pellet from the first tube was resuspended in 250 μl 4X Laemmli buffer. The same 250 μl was transferred to the next tube to resuspend that pellet and so on, giving the “β-ME elution”.

### Acyl-resin-assisted capture

The protocol presented here represents the optimised method, based on a previously published protocol^21^. A schematic workflow of this method is shown in Supplementary Figure 3.

1. A BCA assay was performed to determine the homogenate concentration. The sample was diluted to 2 mg ml−1 with blocking buffer (100 mM HEPES, 1 mM EDTA, 2.5% SDS, pH 7.4) and an aliquot taken (“input”).
2. MMTS was added to 0.5% and the sample incubated at 40 °C for one hour with frequent vortexing.
3. The sample was methanol precipitated three times as above in as few tubes as possible. The final time, the sample was resuspended in 1 ml binding buffer (100 mM HEPES, 1 mM EDTA, 1% SDS, pH 7.4) instead of SB and incubated at 37 °C, 220 rpm for 30 minutes.
4. Meanwhile, 0.25 g thiopropyl Sepharose® 6B beads (Sigma, Dorset, UK) was washed in 20 ml distilled water for 15 minutes. The beads were spun at 3500 rcf, 4 °C for two minutes, the supernatant removed and an equal volume of binding buffer added to the settled slurry.
5. The sample (from step 3) was split into two tubes and 1 ml slurry added to each. An equal volume of 2 M HA pH 7.4 was added to one tube, an equal volume of 2 M Tris.HCl pH 7.4 to the other and a PI tablet added. Samples were incubated on a rocker at room temperature overnight.
6. The following day, samples were spun at 3500 rcf, 4 °C for two minutes and a sample of supernatant was taken (“unbound”). The remaining supernatant was discarded. The bead pellet was washed five times with 5 ml binding buffer, spinning at 3500 rcf, 4 °C for two minutes between washes.
7. Proteins were eluted as in steps 8 and 9 in the ABE protocol above.

### SDS-PAGE

Protein samples were prepared for separation by SDS-polyacrylamide gel electrophoresis (SDS-PAGE) by boiling in Laemmli buffer at 95 °C for five minutes. 15% gels were cast in the Mini PROTEAN 3 system (BioRad, Hemel Hempstead, UK) or pre-cast NuPAGE® 12% or 4–12% Bis-Tris Gels were used (Life Technologies, Paisley, UK). Samples were loaded alongside molecular mass markers using pre-stained protein ladder (Geneflow, Fradley, UK) or SeeBlue Plus2 (Thermo). Gels were run at 180–200 V until the dye reached the bottom of the gel, visualised by staining with Coomassie blue and imaged in a ChemiDoc XRS with Quantity One or ImageLab software (BioRad).

### Western blotting

Proteins were transferred after SDS-PAGE to nitrocellulose submerged in transfer buffer (0.025 M Tris, 0.192 M glycine, 20% methanol) in a BioRad Trans-blot Electrophoresis Transfer Cell, either at 100 V for one hour with an ice pack or 20 V overnight. Nitrocellulose was blocked for one hour in Tris-buffered saline (TBS; 20 mM Tris, 140 mM NaCl, pH 7.4) with 0.1% Tween 20 (TBS-T) and 5% (w/v) dried skimmed milk. The primary antibody was applied at an appropriate dilution in TBS-T supplemented with 5% (w/v) BSA and incubated on a rocker for either one hour at room temperature or at 4 °C overnight. The nitrocellulose was washed three times in TBS-T for five minutes before incubation with anti-mouse-horseradish peroxidase (HRP) or anti-rabbit-HRP (Sigma) for one hour on a rocker. The nitrocellulose was rinsed with TBS-T and visualised using enhanced chemiluminescence (ECL) reagents A (2.5 mM luminol, 400 μM *p*-coumaric acid, 100 mM Tris.HCl pH 8.5) and B (0.018% H2O2, 100 mM Tris.HCl pH 8.5) mixed 1:1, imaged in a ChemiDoc XRS using Quantity One software.

Primary antibodies used were: mouse anti-SNAP-25 and rabbit anti-Munc18 (BD Transduction Laboratories, Ireland); mouse anti-syntaxin 1 (HPC1) and rabbit anti-syntaxin-3 (Synaptic Systems, Göttingen, Germany); rabbit anti-calnexin (Sigma); rabbit anti-VAMP-2 (a gift from M. Takahashi); anti-CSP raised in rabbit as previously described^51^.

### Mass spectrometry

The final eluates were analysed by mass spectrometry. Briefly, the protein extracts were separated using one dimensional SDS-PAGE. Each gel lane was cut into 24 equal slices and digested with trypsin following reduction of any disulphide bonds with DTT and alkylation of free cysteine residues with iodoacetamide. The samples were then transferred into clean sample tubes and centrifuged to remove any debris. The protein digests were placed into glass vials prior to mass spectrometric analysis.

Peptide analysis by liquid chromatography-tandem mass spectrometry (LC-MS/MS) was performed in positive ion mode using a Thermo LTQ-Orbitrap XL LC-MSn mass spectrometer equipped with a nanospray source and coupled to a Waters nanoAcquity ultra performance liquid chromatography (UPLC) system. The samples were initially desalted and concentrated on a BEH C18 trapping column (Waters, Milford, MA, USA). The peptides were then separated on a BEH C18 nanocolumn (1.7 μm, 75 μm × 250 mm, Waters) at a flow rate of 400 nl min−1 using an acetonitrile-water gradient. Spectra were collected using data-dependent acquisition in the range m/z 300–2000 following which individual precursor ions were automatically fragmented using collision induced dissociation (CID).

### Data analysis

The mass spectrometry data were analysed using MaxQUANT (http://maxquant.org/index.htm). Data were searched against a locally implemented MASCOT server (v2.3.01). The initial search parameters allowed for a single trypsin missed cleavage, carbamidomethyl modification of cysteine residues, oxidation of cysteine residues up to trioxidation, oxidation of methionine, N-terminal *N*-acetylation, a peptide mass tolerance of ±10 ppm and a fragment mass tolerance of ±0.8 Da. Peptide charge was +1, +2, +3 and the data were searched against both Swissprot and UniRef, Taxonomy – Rat. The data were searched against IPI rat v 3.68 using PEP as the measure of certainty. Protein identifications were based on at least two peptides per protein, using an FDR of 0.01 for both proteins and peptides. The datasets were processed by removing trypsin and keratin and discarding identifications which have been removed from official databases. Identifications from a single unique peptide were removed from all lists, and any proteins appearing in both control and experimental lists were removed from the experimental list.

### Collation of previous identifications

Palmitoyl-proteins identified with the highest confidence in each previous study using mammalian source material^14–19, 21, 25–33^ (Supplementary Table 1) were collated (Supplementary File 1). Clear orthologues from different species were treated as a common identification. During this collation some proteins were found to no longer be officially recognised, or are known by different names. In all cases the most up-to-date information in the NCBI and UniProt databases were used.

The proportional-area Venn diagram was constructed using eulerAPE (University of Kent, http://www.eulerdiagrams.org/eulerAPE/; v3.0.0).

## Electronic supplementary material

Supplementary Info

Supplementary File 1

Supplementary File 2

Supplementary File 3

Supplementary File 4
