# Supplementary material for: Analysis of the brain palmitoyl-proteome using both acyl-biotin exchange and acyl-resin-assisted capture methods
Source: Sci Rep. 2017 Jun 12;7:3299. doi: 10.1038/s41598-017-03562-7 (PMC5468251; doi:10.1038/s41598-017-03562-7)
Supplement: Supplementary file 1 — Supplementary Info [file 41598_2017_3562_MOESM1_ESM.pdf]

Analysis of the brain palmitoyl-proteome using both acyl-biotin exchange and acyl-resin-assisted capture methods

Matthew J. Edmonds[1], Bethany Geary[2], Mary K. Doherty[2] and Alan Morgan[1*]

[1]Department of Cellular and Molecular Physiology, Institute of Translational Medicine, University of Liverpool, Crown St., Liverpool L69 3BX, UK.

[2]Division of Health Research, University of the Highlands and Islands, Centre for Health Science, Old Perth Road, Inverness IV2 3JH, UK.

Supplementary Information

Supplementary Figure and File captions

Supplementary Figure 1. Identification rate of palmitoyl-proteins. 1819 high confidence palmitoyl-proteins from 16 previous proteomic studies using mammalian tissue (Supplementary Table 1) were collated (Supplementary File 1). The number of studies each palmitoyl-protein was identified in was totalled and plotted.

Supplementary Figure 2. Optimisation of ABE protocol. (A) Rat brain homogenate was treated with MMTS to block free thiols followed by final concentrations of either 1 M HA overnight at room temperature, 20 mM DTT for one hour at 37 °C or 100 mM Tris pH 8.9 for one hour at room temperature. Samples were subjected to Western blotting and probed against CSP. Removal of all its palmitates results in a mass shift from 29 to 22 kDa. (B) Rat brain homogenate was treated with MMTS, followed by HA for the times shown. Protein was recovered by three methanol precipitations before subjecting to Western blotting and probing with anti-CSP. Full depalmitoylation requires an overnight treatment. CSP, cysteine string protein; DTT, dithiothreitol; HA, hydroxylamine; MMTS, methyl methanethiosulphonate.

Supplementary Figure 3. Flow diagram of acyl-biotin exchange methodology. A summary of the experimental process of acyl-biotin exchange is shown, including approximate quantities of material (blue) and points at which samples are removed for subsequent SDS-PAGE and Western blotting analysis (red). The numbers in blue circles refer to the step numbers in the Methods section. β-ME, β-mercaptoethanol; biotin-HPDP, N-[6-(biotinamido)hexyl]-3'-(2'-pyridyldithio)propionamide; CON, control sample; EXP, experimental sample; HA, hydroxylamine; LB, lysis buffer; LB-T, lysis buffer + 0.2% Triton X-100; MMTS, methyl methanethiosulphonate; PI, protease inhibitor; RT, room temperature; SB, solubilisation buffer; SDS-PAGE, sodium dodecyl sulphate polyacrylamide gel electrophoresis.

Supplementary Figure 4. Flow diagram of acyl-resin-assisted capture methodology. A summary of the experimental process of acyl-resin-assisted capture is shown, including approximate quantities of material (blue) and points at which samples are removed for subsequent SDS-PAGE and Western blotting analysis (red). The numbers in blue circles refer to the step numbers in the Methods section. β-ME, β-mercaptoethanol; CON, control sample; EXP, experimental sample; HA, hydroxylamine; LB-T, lysis buffer + 0.2% Triton X-100; MMTS, methyl methanethiosulphonate; PI, protease inhibitor; RT, room temperature; SDS-PAGE, sodium dodecyl sulphate polyacrylamide gel electrophoresis.

Supplementary Figure 5. The relationship between protein identifications from this and previous mammalian palmitoyl-proteomic studies. Palmitoyl-protein identifications from this study were collated along with previous mammalian identifications (Supplementary File 1). The number of studies in which each identification from ABE (A) and acyl-RAC (B) in this study was found was totalled and plotted, along with the totals from all studies (C). (D) A proportional-area Venn diagram showing the overlap between palmitoyl-proteins identified in this and previous studies.

Supplementary Figure 6. Uncropped gel images.

Supplementary File 1. Collated high-confidence palmitoyl-proteins from this and previous proteomic studies. This file is an interactive filterable Excel spreadsheet containing the palmitoyl-proteins identified in all mammalian studies.

Supplementary File 2. Refined list of proteins identified in experimental and control samples for both ABE and acyl-RAC. This file contains the proteins identified across three repeats of ABE and acyl-RAC in both experimental and control samples. Keratin, trypsin and proteins identified from one unique peptide have been removed, and proteins found in both experimental and control lists have been removed from the experimental list.

Supplementary File 3. Complete list of all proteins detected in experimental and control samples for both ABE and acyl-RAC. This file contains all the proteins identified across three repeats of ABE and acyl-RAC in both experimental and control samples based on detection of at least one peptide.

Supplementary File 4. Raw data and ratiometric analysis of label free quantification of all peptides detected in experimental and control samples for both ABE and acyl-RAC. This file contains all proteins identified by ABE and acyl-RAC in both experimental and control samples based on detection of at least one peptide. Sheet 1 shows the complete database. Sheet 2 shows the ratiometric analysis, where a 10:1 ratio of HA:control LFQ intensity is used as the threshold for high-confidence identification. Yellow and green highlighted cells in columns H and I indicate the nine additional proteins identified using this ratiometric threshold compared to the simple cut-off of proteins identified in control samples.

Supplementary Tables

Supplementary Table 1. Findings from previously published palmitoyl-proteomic analyses. All published palmitoyl-proteomic studies to date are listed along with the method used, source material and the reported number of high confidence palmitoyl-proteins identified. Note that there are some discrepancies with the totals indicated in Supplementary File 1 due to removal of some previously predicted proteins from online databases or where they have later been found to be identical to other proteins. 17-ODYA, 17-octadecynoic acid; ABE, acyl-biotin exchange; acyl-RAC, acyl-resin-assisted capture; HUVEC, human umbilical vein endothelial cells; PalmPISC, palmitoyl protein identification and site characterisation; PICA, palmitoyl-cysteine isolation capture and analysis; PPT, palmitoyl-protein thioesterase; SILAC, stable isotope labelling of amino acids in cell culture; SILAM, whole animal stable isotope labelling.

Study	Method	Source material	No. hits	Notes
Ref 12 (Roth *et al.* (2006))	ABE	*Saccharomyces cerevisiae*	47	
Ref 14 (Kang *et al.* (2008))	ABE	rat cultured embryonic neurons and synaptosomes	163	
Ref 29 (Zhang *et al.* (2008))	PICA	HeLa cells	50	probed DHHC2 substrates
Ref 26 (Martin and Cravatt (2009))	click chemistry: 17-ODYA	Jurkat T cells	125	
Ref 19 (Yang *et al.* (2010))	ABE, PalmPISC	DU145 human prostate cancer cells	169	408 total hits from lipid rafts; 112 from rest of membrane; 273 in common
Ref 28 (Yount *et al.* (2010))	click chemistry: alk-16	mouse D2.4 dendritic cells	60	
Ref 16 (Dowal *et al.* (2011))	ABE, PalmPISC	human platelets	215	
Ref 17 (Merrick *et al* (2011))	ABE	RAW 264.7 macrophages	80	
Ref 21 (Forrester *et al.* (2011))	acyl-RAC	bovine brain membranes and HEK293 cells	88	site-specific analysis
Ref 30 (Wilson *et al.* (2011))	click chemistry: alk-16	Jurkat T cells	180	
Ref 25 (Li *et al.* (2012))	click chemistry: 17-ODYA	cultured mouse neuronal stem cells	266	
Ref 27 (Martin *et al.* (2012))	click chemistry: 17-ODYA	mouse T cell hybridomas	415	coupled to SILAC; PPT inhibitors used to study dynamics
Ref 18 (Ivaldi *et al.* (2012))	ABE	human B lymphocytes	95	
Ref 15 (Marin *et al.* (2012))	ABE	human EA.hy 926 endothelial cells	184	
Ref 20 (Hemsley *et al.* (2012))	ABE	*Arabidopsis thaliana*	581	
Ref 31 (Ren *et al.* (2013))	acyl-RAC	mouse adipose tissue and 3T3-L1 adipocytes	856	
Ref 32 (Wan *et al.* (2013))	ABE	mouse brain	378	coupled to SILAM
Ref 33 (Wei *et al.* (2014))	ABE	HUVEC cells	382	coupled to SILAC; effect of insulin stimulation examined
Ref 24 (Foe *et al.* (2015))	click chemistry: 17-ODYA	*Toxoplasma gondii*	501	

Supplementary Table 2. Palmitoyl-proteins identified from ABE. Listed here are identifications passing our criteria and resulting from multiple unique peptides. Proteins identified in previous mammalian proteomic studies are indicated. Detailed mass spectrometry data can be found in Supplementary File 1. PEP, posterior error probability.

Gene	Protein	Peptides	Sequence coverage (%)	Molecular weight (kDa)	PEP	Previously identified?
Uchl1	ubiquitin carboxy-terminal hydrolase enzyme L1	15	92.4	24.838	0	✓
Snap25	synaptosomal-associated protein of 25 kDa	11	69.9	23.315	0	✓
Hbb	haemoglobin subunit β2	14	78.9	15.982	0	
Eno2	γ-enolase	21	66.8	47.14	0	✓
Rap1gds1	RAP1, GTP-GDP dissociation stimulator 1	11	26.7	61.913	0	
Vamp2	vesicle-associated membrane protein 2; synaptobrevin	9	50.9	17.62	0	✓
Eno-ps1	α-enolase	19	43.2	53.923	8.32E-293	
Gmfb	glia maturation factor β	7	43.4	16.863	1.06E-278	
Gdi1	Rab-GDP dissociation inhibitor α	16	39.6	50.536	8.63E-236	✓
Stx1b	syntaxin 1b	13	41	33.244	2.29E-233	✓
Txn1	thioredoxin	7	54.3	11.673	2.92E-219	
Acta1	actin, α skeletal muscle	17	38.2	42.051	1.56E-214	✓
Vamp3	vesicle-associated membrane protein 3; synaptobrevin 3	6	50.5	11.48	3.72E-213	✓
Sh3gl2	SH3-domain growth factor receptor-bound 2 (GRB2)-like 2	15	40.1	52.391	3.83E-195	✓
Ldha	L-lactate dehydrogenase A chain isoform 1	14	40.1	36.45	4.46E-194	✓
Glul	glutamine synthetase	12	35.4	42.267	3.40E-188	✓
Spna2	αII spectrin	54	28.8	285.04	3.17E-185	✓
Ldhb	L-lactate dehydrogenase B chain	12	45.8	36.612	5.85E-178	✓
Cs	citrate synthase, mitochondrial	11	29.6	51.866	4.39E-170	✓
Prdx5	peroxiredoxin 5	14	58.7	22.178	3.66E-168	✓
Asrgl1	L-asparaginase	13	42.6	34.41	2.84E-163	✓
Gstm5	glutathione-S-transferase µ5	7	36	26.629	1.06E-162	✓
Ywhag	14-3-3 protein γ	10	47	28.302	3.57E-161	✓
Glo1	lactoylglutathione lyase	12	62	20.819	4.48E-154	
Gstp1	glutathione-S-transferase P	4	24.8	23.439	4.32E-151	✓
Gfap	glial fibrillary acidic protein	20	43.9	50.07	4.06E-144	
Tagln3	transgelin-3	16	66.3	22.5	7.27E-141	
Gap43	neuromodulin	13	78.8	23.603	2.30E-135	✓
Calb2	calretinin	7	28	31.404	2.01E-125	
Tpm3	tropomyosin α3 chain	23	74.2	29.006	5.98E-122	✓
Map1a	microtubule-associated protein 1A	25	13.1	325.01	3.40E-118	
Ube2n	ubiquitin-conjugating enzyme E2 N	8	56.6	17.124	1.52E-114	✓
Gstm1	glutathione-S-transferase µ1	11	45.7	28.399	1.51E-112	✓
Ppid	peptidyl-prolyl cis-trans isomerase	18	41.1	40.765	5.48E-110	
Ube2l3	ubiquitin-conjugating enzyme E2 L3	10	71.4	17.861	3.92E-109	✓
Hsph1	heat shock protein 105 kDa	11	13.8	96.417	5.49E-103	✓
Eno3	β-enolase isoform 1	6	19.1	47.013	2.38E-101	✓
Mif	macrophage migration inhibitory factor	5	41.7	12.477	4.10E-94	
Stip1	stress-induced phosphoprotein 1	17	37.4	62.569	2.16E-93	✓
Ndufv2	NADH dehydrogenase flavoprotein 2, mitochondrial	3	13.7	27.378	6.77E-87	✓
Ddah2	N(G),N(G)-dimethylaminehydrolase 2	6	28.4	29.688	2.54E-84	
Cnp	2,3-cyclic nucleotide phosphodiesterase	17	50.2	47.268	6.88E-83	✓
Ywhab	14-3-3 protein β	12	48.4	28.054	1.05E-82	✓
Aldoc	fructose bisphosphonate aldolase C	15	46.3	39.283	5.68E-76	✓
Nme2	nucleoside diphosphate kinase B	12	75	17.283	1.02E-75	✓
Sh3gl1	endophilin A1	9	32.6	41.492	7.69E-75	
Rhob	Rho-related GTP-binding protein RhoB	5	40.8	22.123	1.96E-73	✓
Glod4	glyoxolase domain-containing 4	5	20.5	33.267	6.20E-73	
Mdh2	malate dehydrogenase, mitochondrial	10	38.2	35.683	4.17E-70	✓

Gene	Protein	Peptides	Sequence coverage (%)	Molecular weight (kDa)	PEP	Previously identified?
Pcbp1	poly(rC)-binding protein 1	5	17.4	37.497	3.83E-69	
Ppp2r1a	serine/threonine protein phosphatase 2A 65 kDa regulatory subunit A α isoform	16	34.3	65.322	1.48E-68	✓
Rhoa	transforming protein RhoA	4	31.1	21.782	2.21E-67	✓
Hbe1	ε1 globin	2	12.2	16.104	5.32E-67	
Ckmt1	creatine kinase U type, mitochondrial	11	36.1	47.028	5.25E-63	✓
Ndrg2	N-Myc downstream regulated gene 2	9	49.3	40.779	5.80E-61	✓
Snx12	sorting nexin 12	5	27.6	19.807	2.17E-59	
Ola1	Obg-like ATPase 1	9	30.8	44.535	6.00E-57	✓
Scrn1	secernin 1	15	51	46.396	1.84E-54	
Ube2v1	ubiquitin-conjugating enzyme E2 V1	9	49.7	16.355	2.30E-52	✓
Ddt	D-dopachrome decarboxylase	5	59.3	13.133	7.78E-52	
Pafah1b2	platelet-activating factor acetylhydrolase IB subunit β	6	47.6	25.581	4.85E-51	
Trappc3	trafficking protein particle complex 3 (Bet3)	6	35	20.302	2.32E-50	✓
Impact	protein IMPACT	5	26.5	35.995	1.53E-47	
Dbn1	drebrin isoform A	5	12.9	77.471	1.98E-47	
Sirt2	NAD-dependent deacetylase sirtuin 2	7	20.4	43.026	4.56E-47	
Marcks	myristoylated alanine rich C-kinase substrate	8	41.7	30.2	1.15E-45	✓
Pgk1	phosphoglycerate kinase 1	10	34.8	44.538	4.79E-44	✓
Abat	4-aminobutyrate aminotransferase	2	5.4	56.455	6.27E-44	✓
Cltb	clathrin light chain B isoform brain	6	19.2	25.117	5.68E-43	
Vcp	transitional endoplasmic reticulum ATPase	12	20.8	89.348	1.23E-42	✓
Erp29	endoplasmic reticulum protein ERp29	4	17.7	28.574	3.10E-42	
Clic4	chloride intracellular channel protein 4	6	32.8	28.633	4.86E-42	✓
Vim	vimentin	10	26	53.732	1.16E-41	✓
Stx1a	syntaxin 1a	7	35.1	33.067	1.40E-41	✓
Gdi2	Rab-GDP dissociation inhibitor β	5	11	50.537	6.15E-41	✓
Snap91	clathrin coat assembly protein AP180 long isoform	6	11.1	93.753	1.21E-40	✓
Idh3a	isocitrate dehydrogenase [NAD] subunit α, mitochondrial	7	22.1	39.613	1.63E-38	✓
Stxbp1	syntaxin-binding protein 1 (Munc18)	14	37	67.568	3.23E-38	✓
Gstm7	glutathione-S-transferase Yb-3	7	41.3	25.68	3.91E-38	
Mdh1	malate dehydrogenase, cytoplasmic	5	19.8	36.483	7.75E-38	✓
Tubb2a	tubulin β2A chain	6	15.5	49.906	1.48E-37	
Ak1	adenylate kinase isoenzyme 1 isoform 2	6	44.3	21.584	1.17E-35	✓
Map2	microtubule-associated protein 2 isoform MAP2x	3	3	202.41	9.73E-34	
Rac1	Ras-related C3 botulinum toxin substrate 1	7	37	23.436	1.09E-33	✓
Vapa	vesicle-associated membrane protein-associated protein A	8	22.5	27.841	1.33E-33	
Hprt1	hypoxanthine-guanine phosphoribosyltransferase	4	30.3	24.477	1.69E-33	✓
Sfrs3	splicing factor, arginine/serine-rich 3	4	23.2	19.329	2.62E-33	
Hnrnpk	heterogeneous nuclear ribonucleoprotein K	8	19.8	51.028	8.99E-33	✓
Pcbp2	poly(rC)-binding protein 2	4	14.8	38.58	2.73E-32	
Tpt1	translationally-controlled tumour protein	3	20.9	19.462	3.03E-32	✓
Glrx1	glutaredoxin 1	2	8.4	11.879	6.96E-32	
Itpa	inosine triphosphatase	8	57.6	21.927	3.90E-31	
Rps12	40S ribosomal protein S12	3	24.2	14.525	5.63E-31	
Irgq	immunity-related GTPase family Q	8	21.3	59.259	2.33E-30	
Aco2	aconitate hydratase, mitochondrial	9	15.9	85.432	6.49E-30	✓
Hint1	histidine triad nucleotide binding protein 1	2	10.9	21.208	9.81E-30	
Pgam1	phosphoglycerate mutase 1	7	44.1	28.832	1.18E-29	✓
Tpm1	tropomyosin α1 chain	7	26.7	28.704	4.91E-29	
Atp6v1e1	V-type proton ATPase subunit E1	6	28.3	26.128	5.19E-29	

Gene	Protein	Peptides	Sequence coverage (%)	Molecular weight (kDa)	PEP	Previously identified?
Ube2m	ubiquitin-conjugating enzyme E2 M	6	31.1	20.9	1.32E-28	✓
Gsta1	glutathione-S-transferase α3	5	38.9	25.988	1.92E-27	
Dynll2	dynein light chain 2, cytoplasmic	6	39.3	10.35	4.51E-27	
Cnrip1	CB1 cannabinoid receptor-interacting protein 1	4	35.4	18.658	1.43E-26	✓
Cdc42	cell division cycle 42	7	37.7	21.258	1.32E-25	✓
Ube2d3	ubiquitin-conjugating enzyme E2 D3	3	28.8	16.595	1.70E-25	
Arpc3	actin related protein 2/3 complex subunit 3	4	24.7	20.535	2.88E-25	
Cycs	cytochrome c	6	50.5	11.605	4.44E-25	✓
Vapb	vesicle-associated membrane protein-associated protein B	4	12.3	26.916	1.40E-24	
Prdx2	peroxiredoxin 2	7	32.3	21.783	2.32E-24	✓
Reep5	receptor accessory protein 5; deleted in polyposis 1	2	11.6	21.431	1.35E-23	✓
Car2	carbonic anhydrase 2	4	16.9	29.113	1.10E-22	✓
Napg	N-ethylmaleimide-sensitive factor (NSF) attachment protein γ	5	19.2	34.659	2.10E-22	
Eif5a	eukaryotic translation initiation factor 5A-1	5	44.2	16.832	2.37E-22	✓
Ppa1	pyrophosphatase 1	7	24.6	32.771	1.04E-21	✓
Skp1	S-phase kinase-associated protein 1	6	39.3	18.672	1.24E-21	
Tagln2	transgelin-2	7	44.7	22.393	3.84E-21	✓
Ppp1cc	serine/threonine protein phosphatase PP1γ catalytic subunit isoform γ2	2	10.4	38.504	4.87E-21	✓
Khsrp	far upstream element-binding protein 2	6	10.3	74.226	6.16E-21	✓
Akr1a1	alcohol dehydrogenase [NADP$^+$]	2	9.5	36.505	7.11E-21	✓
St13	Hsc70-interacting protein	5	11.4	41.279	8.17E-21	
Pfn1	profilin-1	4	27.1	14.957	1.65E-20	✓
Nras	N-Ras	4	31.7	21.243	1.81E-20	✓
Ncdn	neurochondrin	6	9.1	78.923	2.64E-20	✓
Ube2v2	ubiquitin-conjugating enzyme E2 V2	4	26.9	16.353	2.41E-19	
Bin1	isoform AMPH2-1 of Myc box-dependent-interacting protein 1	4	12.4	64.532	2.75E-19	
Arpc5	actin-related protein 2/3 complex subunit 5	2	31.1	16.32	2.83E-19	
Pitpna	phosphatidylinositol transfer protein α	4	20.7	31.907	3.43E-19	
Dstnl1	destrin-like 1	5	32.7	18.519	4.99E-19	
Hspa9	heat shock 70 kDa protein 9	3	5.2	73.857	1.38E-18	✓
Paip1	poly(A)-binding protein interacting protein 1	3	6.5	42.06	3.89E-17	
Dnajc5	cysteine string protein (CSP)	3	28.8	22.101	4.30E-17	✓
Acp1	low molecular weight phosphotyrosine protein phosphatase, isoform 1	3	26.6	18.151	8.19E-17	
S100b	protein S100-B	3	40.2	10.744	3.36E-16	
Serpina1	α1-antiproteinase	5	15.8	46.135	5.39E-16	
Srprb	signal recognition particle receptor, B subunit	3	3.4	107.22	6.96E-16	✓
Pdia6	protein disulphide isomerise A6	4	12.6	48.76	1.77E-15	✓
Cap1	adenylyl cyclise-associated protein 1	2	8.6	51.588	1.88E-15	✓
Pacsin1	protein kinase C and casein kinase substrate in neurons 1	6	15.6	50.449	2.27E-15	
Ranbp1	RAN binding protein 1	3	15.3	23.596	2.67E-15	✓
Akr1b1	aldose reductase	3	10.4	35.797	3.27E-14	
Slc9a3r1	sodium/hydrogen exchange regulatory cofactor (NHE-RF1)	4	15.4	38.83	1.02E-13	
Alb	serum albumin	4	6.1	69.366	1.28E-13	✓
Nap1l1	nucleosome assembly protein 1-like 1	5	21.7	45.373	1.35E-13	
Gsto1	glutathione-S-transferase ω	4	17	27.669	1.94E-13	✓
Ak2	adenylate kinase 2, mitochondrial isoform 1	4	23.8	26.379	2.73E-13	✓
Dnm1l	dynamin-1-like protein	3	6.4	83.907	3.13E-13	✓
Mog	myelin-oligodendrocyte glycoprotein	2	8.2	27.881	3.35E-13	✓
Gnao1	Gαo G protein subunit	3	9	40.08	3.83E-13	✓

Gene	Protein	Peptides	Sequence coverage (%)	Molecular weight (kDa)	PEP	Previously identified?
Actr3	actin-related protein 3	4	21.8	47.357	1.10E-12	✓
Pfn2	profilin-2	2	14.8	16.114	1.19E-12	
Ahsg	α2-HS-glycoprotein	3	9.8	61.484	1.66E-12	
Gphn	gephyrin isoform 2	3	6.5	83.637	2.54E-12	
Ank2	ankyrin 2 isoform 1	4	1.6	436.89	3.73E-12	✓
Ddah1	N(G),N(G)-dimethylarginine dimethylaminohydrolase 1	4	13	31.426	6.89E-12	
Hdhd2	haloacid dehalogenase-like hydrolase domain-containing 2	3	10.9	42.475	1.57E-11	
Cacybp	calcyclin-binding protein	2	8.7	26.541	1.86E-11	✓
Rpl12	60S ribosomal protein L12	4	25.1	20.91	2.50E-11	✓
Ppp2r5e	protein phosphatase 2, regulatory subunit B, ε isoform	4	12.4	50.346	2.51E-11	
Fth1	ferritin heavy chain	3	15.9	21.126	4.80E-11	
Celf2	CUG triplet repeat RNA-binding protein 2 (CUGBP2)	2	4.1	56.957	7.15E-11	
Eea1	early endosome antigen 1	6	4.2	161.27	8.50E-11	
Sdhb	succinate dehydrogenase [ubiquinone] iron-sulfur subunit, mitochondrial	4	14.2	31.83	9.78E-11	✓
Prdx1	peroxiredoxin 1	5	23.1	22.109	1.00E-10	✓
Pacs1	phosphofurin acidic cluster sorting protein 1	2	2.9	104.7	1.21E-10	
Plp1	myelin proteolipid protein	3	9.7	30.077	1.34E-10	✓
Lypla2	acyl-protein thioesterase 2 (APT2)	3	14.7	24.807	1.36E-10	
Hpcal4	hippocalcin-like protein 4	3	20.9	22.245	2.58E-10	
Hpca	neuron-specific calcium-binding protein hippocalcin	3	10	31.056	3.90E-10	
Got1	aspartate aminotransferase, cytoplasmic; glutamate oxaloacetate transaminase	3	11.4	46.428	4.11E-10	✓
Sri	sorcin	2	10.6	21.624	5.22E-10	
Atp6v1g2	ATPase, H^+ transporting, lysosomal, V1 subunit G2	2	22.9	13.667	5.23E-10	
Bpnt1	3(2),5-bisphosphate nucleotidase 1	2	6.5	33.174	6.36E-10	✓
Isyna1	inositol-3-phosphate synthase 1	3	8.4	60.883	1.54E-09	✓
Ap2b1	AP-2 complex subunit β	3	4.5	105.69	1.57E-09	✓
Sh3glb2	endophilin B2	3	8.4	44.852	1.70E-09	
Pdia3	protein disulphide-isomerise A3	4	11.7	56.623	4.06E-09	✓
S100a16	protein S100-A16	2	16.3	14.224	4.38E-09	
Ppp3ca	serine/threonine protein phosphatase 2B catalytic subunit α isoform isoform 1	4	8.1	58.643	5.46E-09	✓
Gpx1	glutathione peroxidase 1	3	22.4	22.258	1.86E-08	✓
Ppfia3	liprin-α3	4	4.4	149.93	2.31E-08	
Gpi	glucose-6-phosphate isomerise	2	5.7	62.826	3.42E-08	✓
Grb2	growth factor receptor-bound protein 2	3	23	25.206	3.72E-08	
Iah1	isoamyl acetate-hydrolysing esterase 1 homolog	4	18.5	28.004	3.79E-08	✓
Fam49b	family with sequence similarity 49, member B (0910001A06Rik)	2	7.4	36.776	4.98E-08	✓
Eif5	eukaryotic translation initiation factor 5	2	5.6	48.954	1.06E-07	
Nono	non-POU domain-containing octamer-binding protein	2	6.5	54.925	1.19E-07	
Pgls	6-phosphogluconolactonase	3	15.5	30.821	1.52E-07	
Eef1b2	eukaryotic translation elongation factor 1 β2	2	8.9	24.675	1.65E-07	
Nap1l4	nucleosome assembly protein 1-like 4	3	10.2	47.303	1.77E-07	
Atp1b2	β2 subunit of sodium/potassium-ATPase	3	14.7	33.105	1.91E-07	✓
Mt-co2	cytochrome c oxidase subunit II	3	16.3	25.928	2.93E-07	
Cisd1	CDGSH iron sulphur domain 1	2	25.9	12.097	3.13E-07	✓
Gprin1	G protein-regulated inducer of neurite outgrowth 1 (GRIN1)	4	4.3	96.202	5.06E-07	✓
Map1lc3a	microtubule-associated proteins 1A/1B light chain 3A	2	22.3	14.272	5.94E-07	
Ndrg4	brain development-related molecule 1 (NDRG4)	2	11.6	44.319	6.07E-07	

Gene	Protein	Peptides	Sequence coverage (%)	Molecular weight (kDa)	PEP	Previously identified?
Pnpo	pyridoxine-5-phosphate oxidase	2	11.1	30.184	1.18E-06	
Txndc17	thioredoxin-like 5	2	22.8	14.092	1.48E-06	
Gda	guanine deaminase	3	9.7	50.9	2.57E-06	✓
Ckm	creastin kinase M-type	2	10.5	43.044	2.74E-06	
Atp6v1c1	V-type proton ATPase subunit C1	3	10.7	43.9	3.05E-06	
Gnaq	Gαq G protein subunit	2	8.9	42.144	5.20E-06	✓
Pacs2	phosphofurin acidic cluster sorting protein 2	2	2.2	98.028	6.83E-06	
Pura	transcriptional activator protein Pur-α	3	8.1	34.883	7.04E-06	✓
Cyc1	cytochrome c-1	2	7.1	35.434	1.02E-05	✓
Stub1	STIP1 homology and U-box containing protein 1	2	7.2	34.886	1.08E-05	
Tst	thiosulfate sulfurtransferase	2	14.5	33.406	1.24E-05	✓
Atp5o	ATP synthase subunit O, mitochondrial	2	13.6	23.397	1.48E-05	✓
Ppp3r1	calcineurin subunit B type 1	2	19	24.979	1.51E-05	
Homer1	Homer protein homolog 1	2	4.6	41.304	1.81E-05	
RGD1304704	UPF0568 protein C14orf166 homolog	2	7.1	33.824	2.28E-05	
Fetub	fetuin-B	2	6.9	43.169	2.51E-05	
A1bg	α-1B-glycoprotein	3	5.8	56.478	2.77E-05	
Sept7	septin 7 isoform B	2	4.1	50.839	2.95E-05	✓
Serpina3k	serine protease inhibitor A3K	2	7.2	46.561	3.75E-05	
Gga1	Golgi-associated, γ-adaptin ear containing, ARF-binding protein 1	3	8.5	70.008	3.75E-05	
Hnrnpl	heterogeneous nuclear ribonucleoprotein L isoform a	3	11.2	67.902	4.47E-05	✓
Pithd1	PREDICTED: PITH domain-containing protein 1	3	17.3	25.207	4.87E-05	
Usp5	ubiquitin-specific peptidase 5	2	3.3	95.778	7.27E-05	✓
Shroom2	shroom family member 2	2	2.2	165.42	7.43E-05	
Omg	oligodendrocyte myelin glycoprotein	2	4.1	49.58	7.55E-05	
RGD1304693	RCG54799	2	2.7	109.43	7.90E-05	
Hspd1	chaperonin (HSP60)	2	4.3	93.61	9.70E-05	✓
Tceb2	transcription elongation factor B polypeptide 2	2	13.6	13.17	9.91E-05	
Apoa1bp	NAD(P)H-hydrate epimerase	2	9.2	30.89	0.000125	✓
Ccnb1ip1	cyclin B1 interacting protein 1	2	7.6	31.247	0.000131	
Bcas1	breast carcinoma-amplified sequence 1 homolog	2	5.7	63.711	0.000219	
Mobkl3	MPS one binder kinase activator-like 3	2	13.8	26.032	0.000222	✓
Aldh4a1	aldehyde dehydrogenase 4A1	2	6.6	61.868	0.000231	✓
Ipo5	RAN binding protein 5	2	3.4	123.7	0.00029	✓
Rbm28	RNA binding motif protein 28	2	2.8	83.942	0.000334	
Pcdhb12	protocadherin 3	2	3.4	87.473	0.000514	
Flna	filamin-A isoform 1	2	0.9	281.29	0.000592	✓
Epdr1	mammalian ependymin-related protein 1	2	9.8	25.639	0.000721	
Cadps	calcium-dependent secretion activator 1	2	2.4	153.79	0.000734	✓
Aspa	aspartocyalase	2	7.6	35.555	0.000946	
Sgip1	SH3-domain GRB-like (endophilin) interacting protein 1	2	4.4	91.754	0.001399	
RGD1304884	RIKEN cDNA 6430548M08	2	7.1	46.275	0.001691	
Ube2f	NEDD8-conjugating enzyme E2 F	2	23.2	21.08	0.001869	
Ankrd24	ankyrin repeat domain 24	2	1.8	105.59	0.001877	
Utrn	utrophin	2	1.1	392.32	0.003255	✓
Kcnip2	Kv channel interacting protein 2	2	4	26.089	0.004328	

Supplementary Table 3. Palmitoyl-proteins identified from acyl-RAC. Listed here are identifications passing our criteria and resulting from multiple unique peptides. Proteins identified in previous mammalian proteomic studies are indicated. Detailed mass spectrometry data can be found in Supplementary File 1. PEP, posterior error probability.

Gene	Protein	Peptides	Sequence coverage (%)	Molecular weight (kDa)	PEP	Previously identified?
Snap91	clathrin coat assembly protein AP180 long isoform	13	23.9	91.43	1.63E-139	✓
Ckmt1	creatine kinase U-type, mitochondrial	11	39.7	46.961	9.08E-121	✓
Itpa	inosine triphosphatase	10	72.7	21.927	6.01E-111	
Tagln2	transgelin-2	9	56.8	22.393	1.19E-104	✓
Pdia6	protein disulphide-isomerase A6	6	17.1	48.76	1.50E-99	✓
Nme2	nucleoside diphosphate kinase B	10	62.5	17.283	2.30E-99	✓
Cst3	cystatin-C	3	32.9	15.437	1.31E-85	
A1bg	α-1B-glycoprotein	10	22.6	56.478	7.54E-82	
Napg	N-ethylmaleimide-sensitive factor (NSF) attachment protein γ	7	24.4	34.659	1.26E-78	
Acp1	low molecular weight phosphotyrosine protein phosphatase, isoform 1	5	43	18.151	1.18E-73	
Gap43	neuromodulin	8	55.8	23.603	2.97E-66	✓
Pgls	6-phosphogluconolactonase	7	47.8	30.821	3.65E-65	
Pitpna	phosphatidylinositol transfer protein α	12	51.7	31.907	3.25E-61	
St13	Hsc70-interacting protein	5	14.4	41.279	4.83E-54	
Trappc3	trafficking protein particle complex 3 (Bet3)	9	51.7	20.302	1.59E-46	✓
Pdia3	protein disulphide-isomerase A3	8	18.2	56.623	6.72E-42	✓
Gltp	glycolipid transfer protein	7	49.3	23.703	9.74E-42	
Atp5o	ATP synthase subunit O, mitochondrial	7	40.8	23.397	3.38E-41	✓
Omg	oligodendrocyte myelin glycoprotein	7	16.7	49.58	6.71E-40	
Pacs1	phosphofurin acidic cluster sorting protein 1	6	7.1	104.7	1.73E-38	
Ppa1	pyrophosphatase 1	10	43.3	32.771	8.30E-37	✓
Ak2	adenylate kinase 2, mitochondrial isoform 1	5	28.5	26.379	6.13E-33	✓
Lrrc47	leucine rich repeat containing 47	8	27.4	63.53	1.40E-32	✓
Sfrs3	splicing factor, arginine/serine-rich 3	5	28	19.329	1.72E-31	
Celf2	CUG triplet repeat RNA-binding protein 2 (CUGBP2)	3	7.1	56.957	5.70E-30	
Ube2m	ubiquitin-conjugating enzyme E2 M	6	47	20.9	1.21E-29	✓
Agt	angiontensinogen	4	10.5	51.981	2.87E-29	
Pacsin1	protein kinase C and casein kinase substrate in neurons 1	7	20.4	50.449	8.39E-27	
Glod4	glyoxolasae domain-containing protein 4	4	17.4	33.267	1.61E-26	
Prkcsh	glucosidase 2 subunit β precursor	5	9.1	59.218	5.09E-23	✓
Eef1g	elongation factor 1γ	6	16.9	50.06	5.63E-23	✓
Sdhb	succinate dehydrogenase [ubiquinone] iron-sulfur subunit, mitochondrial	6	22	31.83	9.29E-23	✓
Ube2d2	ubiquitin-conjugating enzyme E2 D2	3	28.6	16.735	2.92E-22	
Akr1b1	aldose reductase	4	13.6	35.797	3.05E-22	
Vapa	vesicle-associated membrane protein-associated protein A	6	26.1	27.841	5.64E-22	
Ap1b1	AP-1 complex subunit β1	4	6.6	104.59	1.51E-21	✓
Stub1	STIP1 homology and U-box containing protein 1	7	26	34.886	2.95E-21	
Atp1b2	β2 subunit of sodium/potassium ATPase	5	14.7	33.105	5.73E-21	✓
Canx	calnexin	5	8.6	67.254	6.32E-20	✓
S100a16	protein S100-A16	2	16.3	14.224	7.23E-20	
Nrcam	neuronal cell adhesion molecule long isoform Nc17	6	6.9	143.14	9.39E-20	✓
Ncan	neurocan	6	5.3	136.25	2.11E-19	
RGD1309534	ester hydrolase C11orf54 homolog	5	22.2	34.993	5.81E-19	

Gene	Protein	Peptides	Sequence coverage (%)	Molecular weight (kDa)	PEP	Previously identified?
Ttll12	tubulin tyrosine ligase-like family, member 12	5	12.2	73.897	1.44E-18	✓
Ube2k	ubiquitin-conjugating enzyme E2 K	4	32	22.406	2.15E-18	✓
Serpina1	α1-antiproteinase	6	18.2	46.135	8.42E-18	
Nap1l4	nucleosome assembly protein 1-like 4	5	15.4	47.303	1.04E-17	
Fam49b	family with sequence similarity 49, member B (0910001A06Rik)	4	17.6	36.776	1.91E-17	✓
Csnk2b	casein kinase II subunit β	4	46.5	24.942	6.49E-17	
Syt2	synaptotagmin2	2	5.2	47.209	9.45E-17	✓
Nutf2	nuclear transport factor 2	4	64.6	14.478	6.77E-16	
Map1s	microtubule-associated protein 1S	5	7.9	102.8	7.24E-16	
Serpina3k	serine protease inhibitor A3K	4	17.1	46.561	1.39E-15	
Ndrg4	brain development-related molecule 1 (NDRG4)	4	18.1	44.319	9.06E-15	
Mgll	monoglyceride lipase	2	8.9	33.499	1.85E-14	
Grpel1	GrpE protein homolog 1	2	9.2	24.297	4.19E-14	✓
Rap1gds1	RAP1, GTP-GDP dissociation stimulator 1	4	54.2	14.069	5.29E-14	
Gnao1	Gαo G protein subunit	2	6.8	40.08	6.35E-14	✓
Icam5	intercellular adhesion molecule 5 precursor	5	4.2	133.3	7.61E-14	
Erp29	endoplasmic reticulum protein ERp29	4	15.8	28.574	1.79E-13	
Ranbp1	RAN binding protein 1	3	21.2	23.596	1.89E-13	✓
Git1	ARF GTPase-activating protein GIT1	2	5.5	85.23	3.24E-13	
Txn1	thioredoxin	2	21	11.673	3.73E-13	
Rpl12	60S ribosomal protein L12	3	17.3	20.91	8.13E-13	✓
Aspa	aspartocyalase	4	15.3	35.555	8.25E-13	
Apoa1bp	NAD(P)H-hydrate epimerase	5	23	30.89	1.95E-12	✓
Mog	myelin-oligodendrocyte glycoprotein	2	10.2	27.881	1.04E-11	✓
Gls	glutaminase kidney isoform	4	7.7	74.023	1.39E-11	✓
Cndp2	cytosolic non-specific dipeptidase	4	12.8	52.693	1.67E-11	✓
Mapk3	mitogen-activated protein kinase 3	2	7.4	45.769	4.66E-11	
Tpt1	translationally-controlled tumour protein	2	15.1	19.462	7.73E-11	✓
Slc4a10	sodium-driven chloride bicarbonate exchanger	3	4.4	125.64	8.36E-11	
Bcan	Brevican core protein	2	3.3	96.056	2.45E-10	
Ly6h	lymphocyte antigen 6 complex, locus H	2	10.7	20.781	2.46E-10	
Map1lc3a	microtubule-associated proteins 1A/1B light chain 3A	3	22.3	14.272	4.73E-10	
Lypla1	acyl-protein thioesterase 1 (APT1)	2	17	24.708	6.37E-10	✓
Hdhd2	haloacid dehalogenase-like hydrolase domain-containing 2	2	7.6	42.475	6.79E-10	
Thy1	Thy-1 membrane glycoprotein precursor	3	22.4	18.172	1.11E-09	✓
Hspa5	78 kDa glucose-regulated protein (GRP-78)	2	3.8	72.346	1.17E-09	✓
Vapb	vesicle-associated membrane protein-associated protein B	2	11.1	26.916	1.49E-09	
Nol3	nucleolar protein 3	2	12.2	24.576	1.91E-09	
Nefl	neurofilament light polypeptide	2	3.5	61.335	2.68E-09	✓
Ndufab1	NADH dehydrogenase (ubiquinone) 1, α/β subcomplex 1	2	9.6	17.514	2.88E-09	
Atp6v1g2	ATPase, H^+-transporting, lysosomal, V1 subunit G2	2	22.9	13.667	4.41E-09	
Psmb2	proteasome subunit β type 2	2	14.9	22.912	5.58E-09	✓
Pnpo	pyridoxine-5-phosphate oxidase	2	11.1	30.184	5.99E-09	
Clta	isoform Brain of clathrin light chain A	4	13.3	26.98	6.90E-09	
Stk39	STE20/SPS1-related proline-alanine-rich protein kinase	2	8.1	60.05	8.21E-09	
Nfasc	neurofascin	4	6.7	138	1.45E-08	✓
Pgp	pyridoxal (pyridoxone, vitamin B6) phosphatase	3	20.1	44.182	2.44E-08	
Cadps	calcium-dependent secretion activator 1	3	3.2	152.88	3.98E-08	✓
Hspa9	heat shock 70 kDa protein 9	3	5.3	73.857	5.13E-08	✓
Pak1	serine/threonine protein kinase PAK1	4	10.5	60.577	5.14E-08	

Gene	Protein	Peptides	Sequence coverage (%)	Molecular weight (kDa)	PEP	Previously identified?
Necab2	neuronal calcium binding protein NECAB2	3	13.4	39.444	6.07E-08	
Cyb5b	cytochrome b5 type B	2	28.1	16.265	6.49E-08	✓
Ipo4	importin 4	3	3.7	119.09	7.28E-08	
Cygb	cytoglobin	2	17.4	21.496	1.02E-07	
Ndufb9	NADH dehydrogenase (ubiquinone) 1, β subcomplex 9	3	20.7	21.892	1.06E-07	
Scarb2	scavenger receptor class B member 2; CD36 antigen-like 2; lysosomal integral membrane protein 2 (LIMP-II)	3	9.4	54.09	1.19E-07	✓
Psma1	proteasome subunit α type 1	4	14.1	29.517	1.44E-07	✓
Fuom	fucose mutarotase	2	20.3	16.83	2.05E-07	
Pacs2	phosphofurin acidic cluster sorting protein 2	3	3.3	98.028	2.52E-07	
Plcb1	1-phosphatidylinositol-4,5-bisphosphate phosphodiesterase β1	3	3	138.34	2.75E-07	✓
Ppa2	pyrophosphatase (inorganic) 2	2	7.3	37.842	3.76E-07	✓
Txndc17	thioredoxin-like 5	3	21.1	14.092	4.19E-07	
Fbxl15	F-box and leucine-rich repeat (LRR) protein 15	2	16.3	33.179	8.18E-07	
Hnrnpab	CArG-binding factor A	2	8.1	36.232	8.81E-07	
Fam98b	family with sequence similarity 98, member B	2	10.4	45.513	1.32E-06	
Anp32b	acidic leucine-rich nuclear phosphoprotein 32 family member B	3	12.8	37.038	1.38E-06	
Fxyd7	FXYD domain-containing ion transport regulator 7	2	20	8.4867	1.48E-06	
Akr1b8	aldose reductase-like protein	2	5.1	36.19	1.60E-06	
Vta1	Vps20-associated 1 homolog (*S. cerevisiae*)	2	12.3	33.975	2.77E-06	
Fetub	fetuin-B	3	13.2	41.532	3.02E-06	
Cmpk1	UMP-CMP kinase	2	8.8	25.833	3.54E-06	✓
Gbas	glioblastoma amplified sequence	2	10	32.941	5.27E-06	
Ado	2-aminoethanethiol (cysteamine) dioxygenase	2	14.8	28.448	5.54E-06	
Uchl3	ubiquitin carboxy-terminal hydrolase isozyme L3	2	14.8	26.123	6.48E-06	
Ube2f	NEDD8-conjugating enzyme E2 F	2	17.8	21.08	6.64E-06	
Atox1	copper transport protein ATOX1	2	32.4	7.2924	8.66E-06	
Snx12	sorting nexin 12	2	10.6	19.807	9.95E-06	
Plxnb1	plexin B1	3	2.3	231.81	1.37E-05	
Anp32a	acidic leucine-rich nuclear phosphoprotein 32 family member A	2	15	28.564	2.03E-05	
Ctnna2	catenin α2	2	5.7	105.28	2.18E-05	
Eif4g1	eukaryotic translation initiation factor, γ1 isoform a	2	1.1	175.7	2.71E-05	
Basp1	brain acid soluble protein 1	2	16.3	21.817	2.74E-05	
Akap7	A kinase (PRKA) anchor protein 7	2	5.7	39.417	3.92E-05	✓
Hist1h2bm	histone H2B type 1-M	2	16.3	16.352	4.11E-05	
Fis1	mitochondrial fission 1 protein	2	17.1	16.994	4.17E-05	
Khdrbs1	KH domain-containing RNA-binding signal transduction-associated protein 1	2	7.7	48.315	5.03E-05	✓
Ddah1	N(G),N(G)-dimethylarginine dimethylaminohydrolase 1	3	21.1	31.426	7.62E-05	
Rbm28	RNA binding motif protein 28	2	2.8	83.942	0.000136	
Eif3e	eukaryotic translation initiation factor 3 subunit E	2	3.8	52.22	0.000141	✓
Ptges3	prostaglandin E synthase 3	2	10.6	18.721	0.0002	
Ufc1	Ufm1-conjugating enzyme 1	2	19.8	19.492	0.000238	✓
Sod2	superoxide dismutase [Mn], mitochondrial	2	19.4	24.674	0.000375	✓
MGC94207	similar to Riken cDNA C030006K11	2	12.8	24.397	0.000497	
Eef1b2	eukaryotic translation elongation factor 1 β2	2	7.1	24.675	0.000694	
Nrn1l	neuritin 1-like	2	8	17.429	0.001057	
Hpx	hemopexin	2	3.7	51.35	0.001378	

Gene	Protein	Peptides	Sequence coverage (%)	Molecular weight (kDa)	PEP	Previously identified?
Ppp2r4	protein phosphatase 2A, regulatory subunit B	2	12.1	36.617	0.001659	✓
R3hdm	R3H domain containing 1	2	1.6	124.12	0.003365	
Ppp2ca	serine/threonine protein phosphatase 2A catalytic subunit α isoform	2	14.6	35.608	0.004665	✓
RGD1311595	RGD1311595 protein	2	1	225.38	0.004687	
Rps6ka2	ribosomal protein S6 kinase α2	2	10.2	18.787	0.00572	

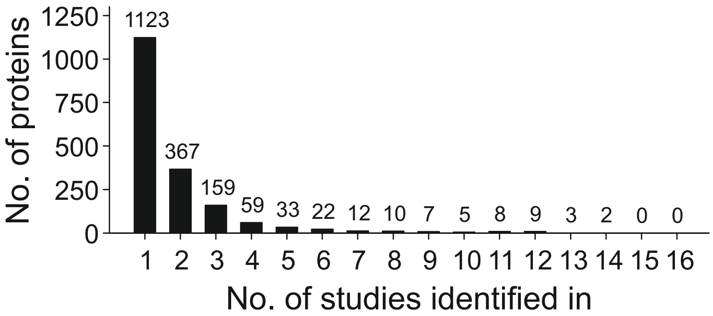

Supplementary Fig 1 Edmonds et al

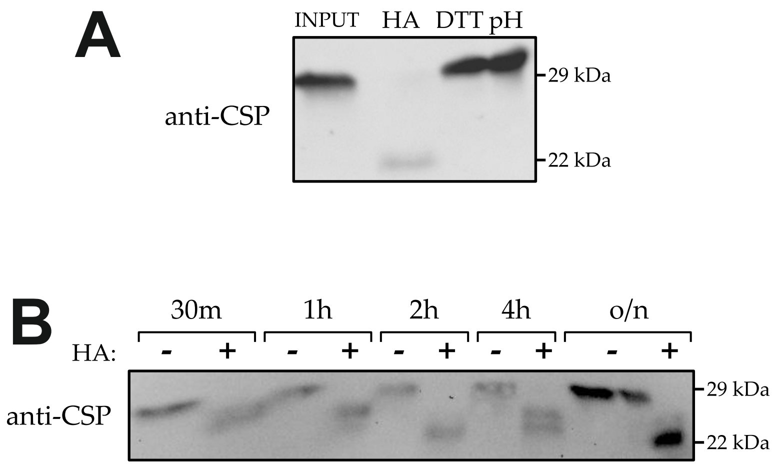

Supplementary Fig 2 Edmonds et al

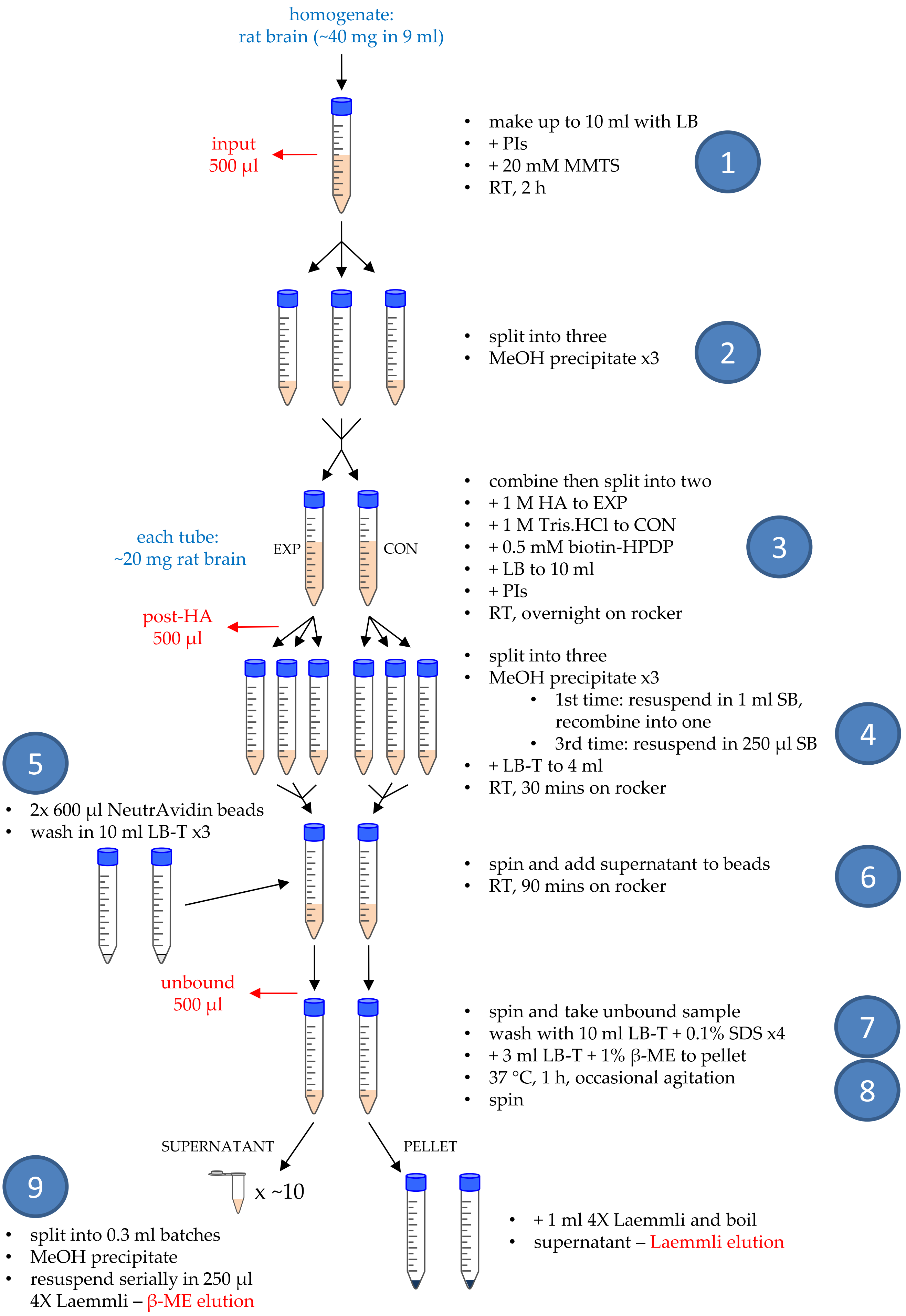

Supplementary Fig 3 Edmonds et al

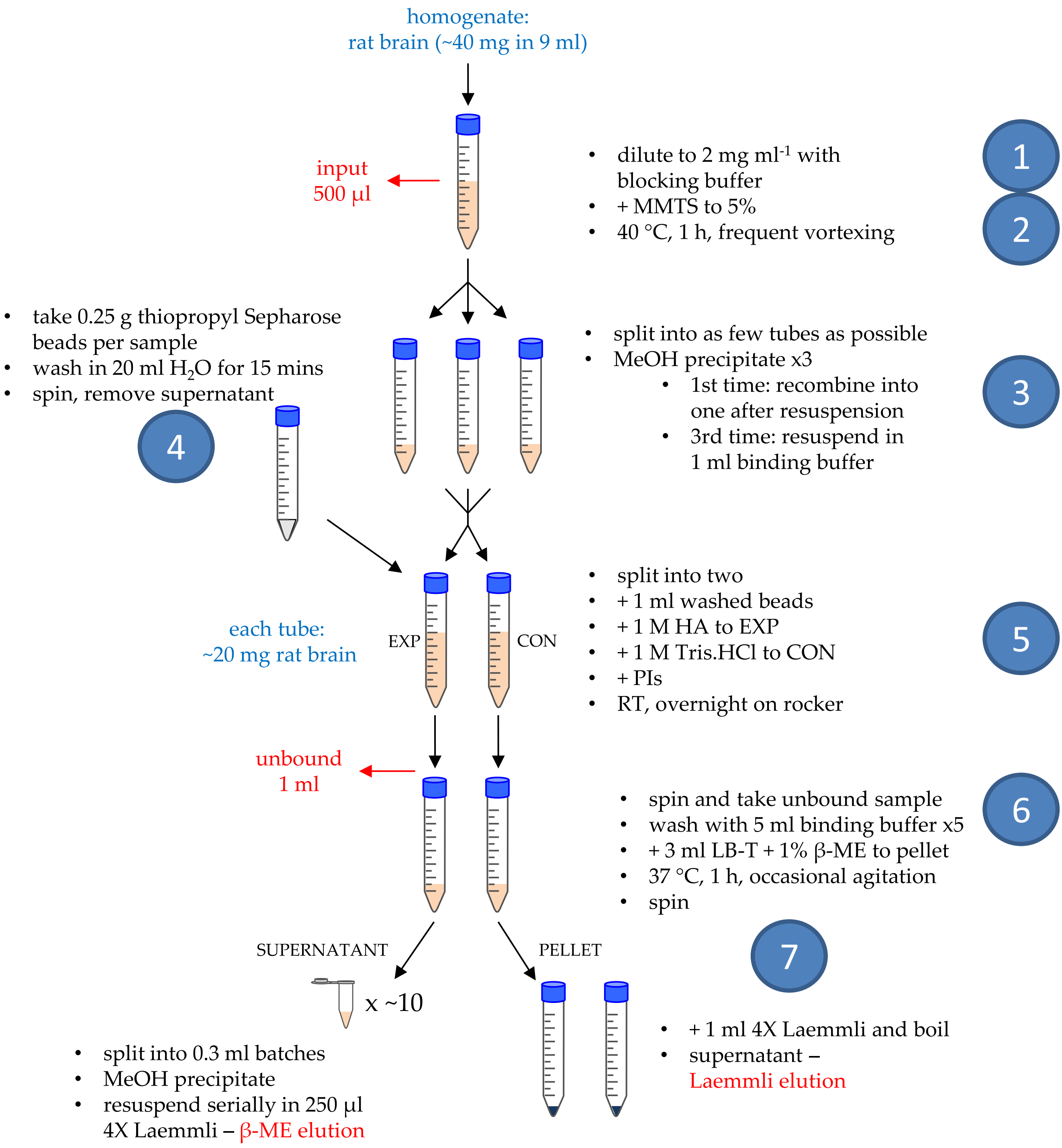

Supplementary Fig 4 Edmonds et al

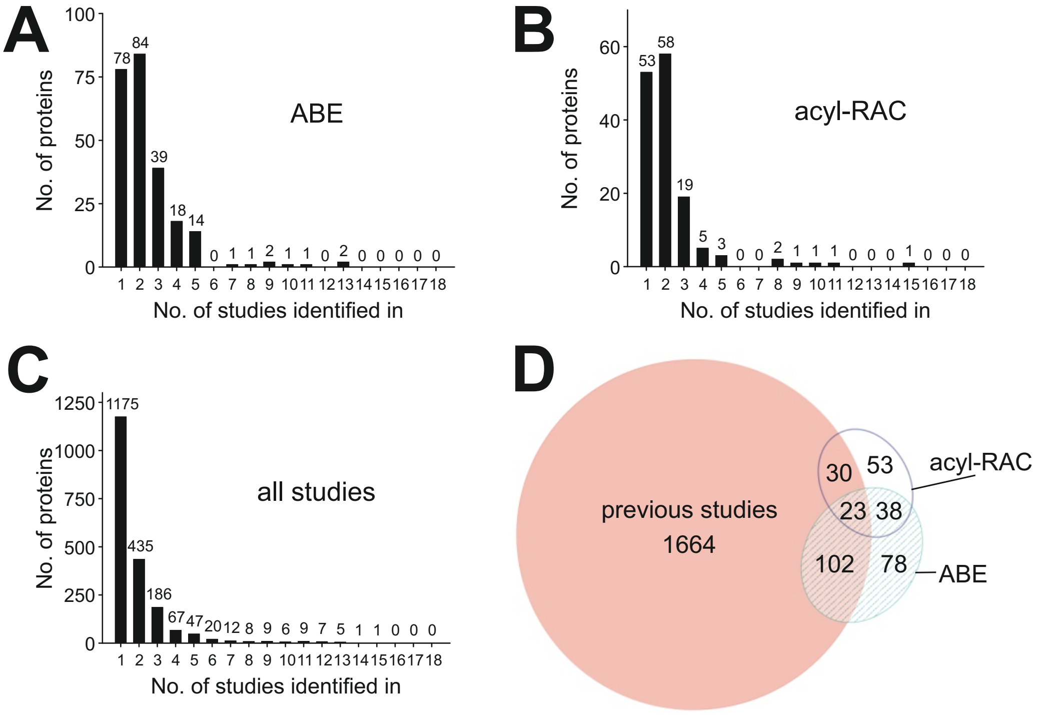

Supplementary Fig 5 Edmonds et al

Figure 2A - CSP

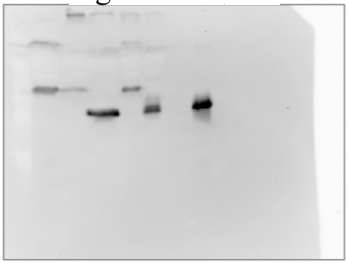

Figure 2B - CSP

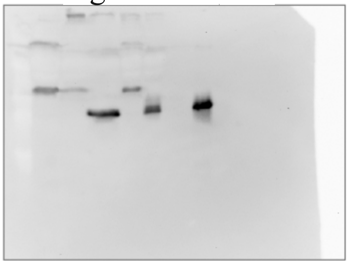

Figure 2B - SNAP-25

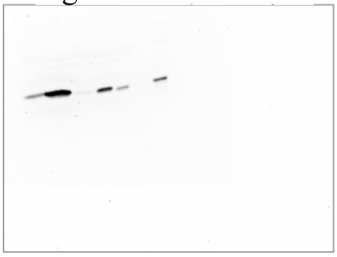

Figure 2B - VAMP-2

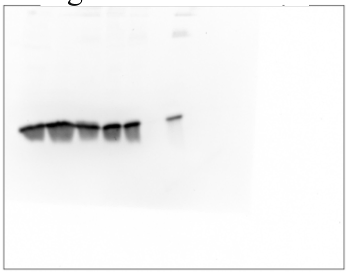

Figure 2B - syntaxin-1

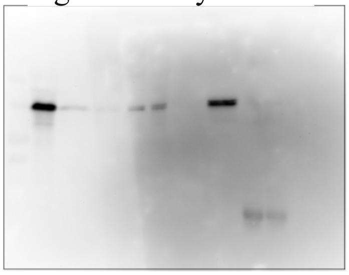

Figure 2B - syntaxin-3

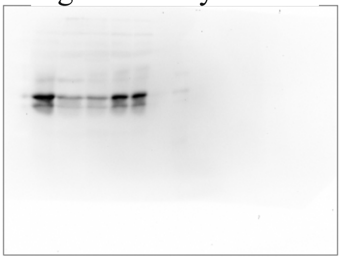

Figure 2C - stained gel

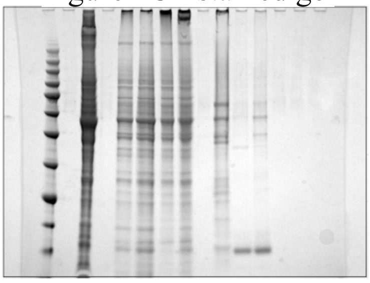

Figure 3A - stained gel

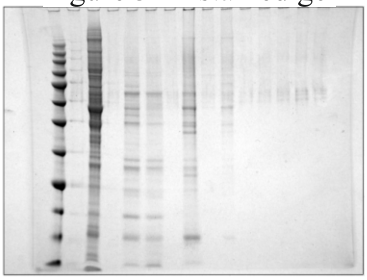

Figure 3B - stained gel

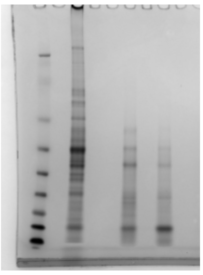

Figure 3C - calnexin

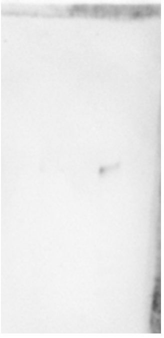

Figure 3C - CSP

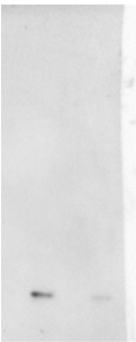

Figure 3C - VAMP-2

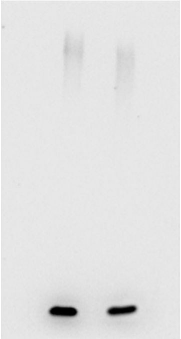

Supplementary figure 2A - CSP

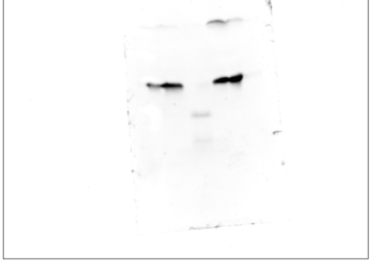

Supplementary figure 2B - CSP

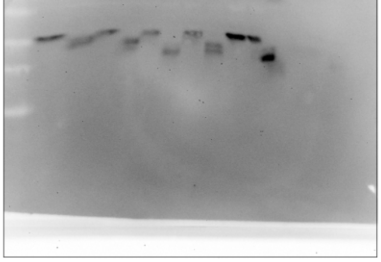

Supplementary Fig 6 Edmonds et al
